# Beyond the brain: a computational MRI-derived neurophysiological framework for robotic conscious capacity

**DOI:** 10.1016/j.neubiorev.2025.106430

**Published:** 2025-10-18

**Authors:** Álex Escolà-Gascón, Kenneth Drinkwater, Andrew Denovan, Neil Dagnall, Julián Benito-León

**Affiliations:** a Department of Quantitative Methods and Statistics, Comillas Pontifical University, established by the Holy See, Vatican City State; b Faculty of Health, Psychology and Social Care, Manchester Metropolitan University, Manchester, United Kingdom; c School of Psychology, Liverpool John Moores University, Liverpool, United Kingdom; d Department of Neurology, 12 de Octubre University Hospital, Madrid, Spain; e Group of Neurodegenerative Diseases, Hospital Universitario, 12 de Octubre Research Institute Madrid, Spain; f Network Center for Biomedical Research in Neurodegenerative Diseases, Madrid, Spain; g Department of Medicine, Faculty of Medicine, Complutense University, Madrid, Spain

**Keywords:** Attribution Consciousness Index, Disorders of consciousness, Generative artificial intelligence, Anesthesia monitoring, Robotic consciousness

## Abstract

Explaining when neural activity supports conscious processing remains an unresolved question in neuroscience. Current frameworks describe correlates of consciousness but rarely provide thresholds to predict its emergence or recovery. We introduce the *Attribution Consciousness Index* (ACI), a metric that estimates the generative potential of consciousness by balancing measures of dynamic information (Φ) and complexity (κ) expressed as a normalized odds ratio. Using the empirically validated Connectome-76 within The Virtual Brain, we ran 500 resting-state simulations, selecting lowest-entropy regions to capture informative subnetworks. The ACI followed a log-normal distribution and highlighted hubs—cingulate cortex, dorsomedial prefrontal cortex, hippocampus, and amygdala—implicated in conscious processing. To test generality, we extended the framework to an artificial neural architecture with hierarchical modules, nonlinear Hebbian plasticity, and controlled entropy. Across 1921 executions, the ACI conformed to log-normal laws, enabling robust thresholding. Kernel ridge regression showed predictive validity: AI-derived ACI patterns explained 38.4 % of variance in human ACI distributions, revealing transferable principles between biological and artificial circuits. This extension indicates that ACI can guide artificial-consciousness models implementable in robotics, providing measurable criteria for when robotic systems might sustain conscious-like states. Two contributions are novel. First, ACI thresholds provide interpretable decision points: values above 10 correspond to probabilities greater than 90 % for conscious emergence. Second, the framework offers translational applications—from prognosis in disorders of consciousness, anesthesia monitoring, and neurorehabilitation to evaluating neuroprosthetics, generative AI, and robotics with conscious capacities. While ACI does not measure subjective experience, it predicts when neural or artificial conditions are poised to sustain it.

## 1. Introduction

A connectome is a dynamic, operational map of brain regions that captures the intricate web of connections and neural circuits sustaining essential functions (Huang et al., 2021). While some connectomes remain hypothetical or depend exclusively on computational simulations, others have been rigorously validated in biological systems and are now widely deployed across diverse species (Nern et al., 2025; Winding et al., 2023). Consider, for example, efforts to enhance visual acuity by targeting specific neural networks: empirically grounded connectomes provide researchers with a powerful framework to test whether an intervention modulates the optic nerve’s visual pathways—without compromising human health or incurring prohibitive costs (Boyd, 2024). In such contexts, connectomes offer precisely controlled virtual environments where experimental effects can be replicated and theoretical models refined, thereby strengthening the foundations of conventional research (Jamison et al., 2025). Neither neuroscience nor engineering should underestimate the transformative potential of empirically validated connectomes. In the least favorable scenario, they may yield inconclusive or flawed models (Vassallo et al., 2024). Yet in the most promising cases, they can evolve into comprehensive atlases capable of illuminating some of the most enduring scientific mysteries—among them, the origins of consciousness and our remarkable capacity for subjective experience (Chalmers, 1995; Lenharo, 2024). As artificial intelligence and quantum computing advance at an unprecedented pace, these questions have acquired new urgency, prompting us to ask whether machines themselves could one day develop self-awareness (Escolà-Gascón, 2025; Escolà-Gascón and Benito-León, 2025).

## 2. How to assess the degree of consciousness in AI

There are two main meta-theoretical frameworks for addressing how consciousness can be evaluated in artificial intelligence. The emergent approach holds that consciousness arises from the informational complexity of physicochemical processes within systems—particularly the brain—rather than constituting a fundamental property of the universe or an entity separate from it (Bunge, 1977). This view suggests that the intricate organization of biological structures enables perception and self-recognition (Santos, 2025). In this framework, consciousness is not simply a byproduct but an emergent property of highly ordered systems, supporting the hypothesis that sufficiently advanced artificial intelligence might eventually develop a sense of identity and awareness of the information it processes (Majorek, 2012).

By contrast, materialist reductionism argues that consciousness is solely the deterministic outcome of physicochemical processes operating within neural systems (Foss, 1995). From this perspective, conscious experience is confined to biological organisms with a central nervous system (Miller et al., 2025). Yet even within reductionist paradigms, there is room for more nuanced positions. Some materialist accounts allow that artificial consciousness could emerge, provided computational architectures faithfully reproduce the material dynamics underpinning conscious states (Martínez-Pernía et al., 2025). Biological naturalism, articulated by Anil Seth (2025), exemplifies this conditional openness, though he also underscores that such scenarios remain unlikely. It is important to note that this synthesis intentionally excludes dualist and metaphysical interpretations—not to discount their relevance, but because they fall beyond the scope of the present discussion. Both emergent and materialist paradigms remain essential, as any rigorous evaluation of consciousness requires not only data but also coherent conceptual frameworks and precise definitions (Wagner et al., 2021). Without clarity about what consciousness is, no measurement can claim genuine validity.

Building on the principles of computational emergentism, numerous models have been developed to investigate the plausibility of machine consciousness. In general, these assessments take the form of cognitive or optimal-performance evaluations, often referred to as *C-Tests*, which are designed to gauge specific sensory capabilities of artificial intelligence systems as they engage in learning and decision-making tasks (Bayne et al., 2024). A central limitation inherent to all such approaches is their inability to discriminate between the mere simulation of certain abilities and the genuine, voluntary use of sensory information that the system is intended to integrate. Nevertheless, these measurement frameworks arise from theoretical paradigms that seek to elucidate the origins, mechanisms, and outcomes of conscious experience. The most prominent examples include *Integrated Information Theory* (IIT), which evaluates a system’s capacity to integrate information (Tononi, 2004, 2005); *Higher-Order Theories* (HOT) which examine the ability to generate metarepresentations (Seth, 2025); and *Global Workspace Theory* (GWT), which, although it shares the materialist foundations, focuses on how widespread neural activation may give rise to phenomenological experience (Baars, 2005; Baars et al., 2021).

## 3. Information and complexity in consciousness

Although a recent review by Kuhn (2024) identified more than 200 theories seeking to resolve the question of what consciousness is and how it operates, scientific research has largely concentrated on two major theoretical frameworks. The first, IIT, adopts a computational, systemic, and phenomenological approach, which is firmly situated within the emergent paradigm of consciousness (Negro, 2022). The second, GWT, is essentially cognitive and inductive, and can be positioned within the materialist reductionist perspective (Baars, 2005; Baars et al., 2021). Rather than detailing the specific tenets of these two leading theories here, in light of the report’s objectives, it is more relevant and necessary to focus on the aspects they share in common.

Both IIT and GWT converge on the same unit of analysis: the information being processed. IIT holds that conscious experience arises when the system integrates information. This framework posits that integration is the point at which subjective interpretation, inference, and the construction of feelings occur (Cea and Signorelli, 2025). In contrast, GWT posits that conscious experience arises from the activation of multiple brain structures that integrate information into a composite representation, known as experience (Baars et al., 2021). Typically, evaluations of when artificial intelligence might develop consciousness are grounded in one of the two paradigms already mentioned—emergent or reductionist—and are implemented in practice through either IIT or GWT models. Rather than regarding them as mutually exclusive, we propose assessing or measuring when AI could develop consciousness by combining properties drawn from both IIT and GWT (Cogitate et al., 2025).

In essence, IIT and GWT share two properties that have so far received little attention: information and complexity. In this respect, both theories articulate their explanations of consciousness through informational units and system complexity, uniting them under a functionalist materialist framework (D’Angiulli and Sidhu, 2025). It is true that higher-order theories of consciousness also include these elements, though they typically emphasize them within the context of computational functionalism (Seth and Bayne, 2022). In IIT, information operates at an intrinsic level, with the system understood to organize information internally through causal relational structures. Complexity manifests in both the system’s dynamic changes and in measures of integration that quantify the extent to which information is unified (Albantakis et al., 2023).

This is in contrast to GWT, where information operates at a distributed and global level, where mental contents enter a common workspace from which they can be broadcast to multiple subsystems in the brain. Here, complexity arises in determining which brain structures are active at any given moment to utilize that information in composing a functional and conscious experience (Farisco and Changeux, 2023).

Computational functionalism underlies both perspectives, as it views information and complexity as abstract operations that, in principle, can be implemented by any suitable physical system, without requiring any specific ontological commitment to the substrate. However, as Anil Seth (2025) has cautioned, the problem with computational functionalism is that it tends to ignore the experiential, or phenomenological, aspect of consciousness by reducing it to a set of syntactic transformations, without guaranteeing that such transformations will give rise to consciousness in its strongest sense.

Why do contemporary research programs in consciousness tend to focus on emergent or materialist perspectives, rather than exploring the mathematical potential inherent in the two properties that are information and complexity? Perhaps it is because most current approaches remain captive to epistemological frameworks that privilege the observable and functional over the ontological and structural, leaving deeper and more rigorous avenues for integrating experience, form, and mathematics largely unexplored.

## 4. Analogical framework for consciousness

To achieve this productive integration of informational units and system complexity, we propose drawing on the concept of *analogia entis* (the “analogy of being”), which underpins the Neo-Thomist paradigm concerning the existence of reality (Nemes, 2022). Neo-Thomism emerged as one of the rationalist frameworks that accompanied the rise of scientific modernism in the late nineteenth century (Polak and Rodzeń, 2022). Its promulgation was led by Pope Leo XIII, who argued persuasively that faith and science are not adversaries but collaborators, united through both scientific method and rational inquiry (Gummess, 2025). As a demonstration of this conviction, he inaugurated the *Vatican Observatory* at Castel Gandolfo in 1891.

Our aim here, however, is not to recount the historical development of Neo-Thomism, but rather to clarify how the rational tool of the analogy of being may help advance the contemporary question of when artificial intelligence could begin to attain self-awareness. The analogy of being is a logical framework showing that the existence of phenomena does not occur solely in a univocal sense (identical in all cases) nor an equivocal sense (completely different in each instance) but also manifests analogically—seeking to preserve unity amid the diversity of reality (Salas, 2009). In this context, “analogically” does not refer to the rhetorical figure of metaphor or analogy in the contemporary argumentative sense. Instead, it describes a self-similar relation established to maintain an ontological balance between identity and difference (Dompere, 2024).

From a logical perspective, materialist reductionism is closely aligned with the univocal view of consciousness. At the same time, the emergent approach resonates with the notion that consciousness is equivocal or variable, depending on how it emerges in each case. Yet Neo-Thomist philosophers demonstrated that univocity and equivocity alone are insufficient to account for how entities exist and function (Dvořák, 2010). For this reason, they proposed the analogy of being as a model of proportional participation. According to this logic, analogy serves as a relational structure that enables the proportional and comparative assessment of entities’ ontologies, taking into account both their similarities and differences. In our case, we will focus on identifying degrees of differentiation, aiming to determine what level of proportion or ratio an analogy must reach to create a rupture from which a new entity might emerge. To clarify this idea in precise mathematical terms, we present below a formal operational formulation of the analogy of being, upon which we will define the hypotheses and objectives of this study.

Let E be the set of all beings (or entities) under consideration; $e_{i}\in E$ denotes a being $e_{i}$ within the set. Each $e_{i}\in E$ participates in being to a certain degree, represented by a function p:E→[0,1], where 1 represents full being (*actus purus*) and 0 represents non-being (or pure privation). The principle of univocity would assert that for all $e_{i}$ in the set, denoted as $e_{j}\in E$, their degree of being is identical: $p\left(e_{i}\right)=p\left(e_{j}\right)$. This implies no ontological diversity and collapses all beings into a single mode—a view that fails to account for the variety of modes of being found in reality. Conversely, equivocity would mean that for $e_{j}\in E$, their degrees of being are semantically incomparable, i.e., $p\left(e_{i}\right)$ and $p\left(e_{j}\right)$ have no meaningful relational mapping. This renders all discourse about “being” meaningless across different entities. To overcome these limitations, *analogia entis* introduces a logic of proportional participation: for some $e_{i}\neq e_{j}$, we have $p\left(e_{i}\right)\neq p\left(e_{j}\right)$, yet both lie within the open interval [0, 1], meaning they share in being in proportionally different ways. The analogical ratio between any two beings is given by: $A\left(e_{i},e_{j}\right):=|p\left(e_{i}\right)/p\left(e_{j}\right)|$, assuming that $p\left(e_{j}\right)\neq 0$. This quantifies the ontological proportionality between them.

Additionally, we define an analogical metric: $d_{A}\left(e_{i},e_{j}\right):=\left|p\left(e_{i}\right)-p\left(e_{j}\right)\right|$, which enables us to view E as a topological space structured by varying degrees of being. Crucially, the *transitivity of analogy* allows these proportional relations to formulate as $p\left(e_{i}\right)/p\left(e_{k}\right)\approx \left[p\left(e_{i}\right)/p\left(e_{j}\right)\right]\cdot \left[p\left(e_{j}\right)/p\right.\left.\left(e_{k}\right)\right]$. This reflects how analogy can propagate across ontological comparisons without collapsing into identity. Finally, the ordering of being is formalized: if $p\left(e_{i}\right)>p\left(e_{j}\right)$, then we denote $e_{i}>e_{j}$, meaning that $e_{i}$ has ontologically more actuality than $e_{j}$. Thus, the *analogia entis* provides a rigorous mathematical framework to structure being as proportionally distributed, capturing the intuition that things “are” in different but comparable ways—something that neither univocity nor equivocity can do coherently. This logical, formal, and mathematical foundation of the analogy of being will serve as the basis for the approach, equations, and analyses presented in the following sections.

## 5. Foundational assumptions

If we understand that consciousness may be a state permitted or emergent in artificial systems, the question that scientists interested in this issue must ask is both basic and profound: from which artificial systems should we expect any degree of consciousness to arise? While researchers are indeed exploring how to identify or induce consciousness in artificial intelligences, there is no scientific roadmap delineating how this search should proceed or under what conditions it should be conducted. In this sense, searching for consciousness in AI is akin to looking for the smallest needle in the largest haystack in the universe.

Suppose we expect to make progress purely through trial and error—relying solely on uncertain trust or hope that we will eventually detect some form of consciousness in AI—as Anil Seth (2025) concluded. In that case, the prospects are far from optimistic. This highlights a need that few scientists have seriously addressed: how can we recognize which artificial systems are most likely to manifest consciousness? To describe the measurement of this recognition, we refer to it as the *generative potential of artificial consciousness*. Determining the extent to which we can identify systems with the potential to exhibit any degree of consciousness is the first step we must take as scientists, before attempting to define evaluation scales for the cognitive functions an AI might display. Considering the properties shared by IIT and GWT—specifically, information and complexity—our aim in this report is to mathematically define a new system of equations that enables us to apply the formal logic of the analogy of being, including transitivity, to the measurement of informational levels and system complexity, without disregarding the phenomenological dimension of conscious experience.

We hypothesize that the emergence of consciousness within an artificial system depends on the proportional relationship between two key properties: (1) the amount of structured information the system generates over time, and (2) the level of dynamic complexity inherent in its activity. Consciousness is more likely to emerge when the system produces a high amount of information in a proportionally stable and coherent manner—that is, when informational richness is sustained without excessive instability or noise across temporal or spatial scales. Therefore, we mathematically state this hypothesis in the following terms:

- E: the expected probability of consciousness emergence.
- $C_{d}$: a measure of dynamic complexity, reflecting the ratio between generative activity and signal variability.
- $H_{i}$: a measure of internal informational coherence or self-similarity across regions, levels, or transformations of the signal.
- g(x): the generativity function (e.g., absolute value of the first derivative of signal R(t)).
- v(x): the variability function (e.g., absolute value of the second derivative of R(t)).
- ω: the domain over which the signal is defined (e.g., time).

Accordingly, we propose the following general transitivity relation in Eqs. (1) and (2):

(1) $$H_{0}:P\left(E=1|C_{d},H_{i}\right)=P(E=1)\;H_{1}:P\left(E=1|C_{d},H_{i}\right)=F\left(C_{d}\cdot H_{i}\right),\frac{dF}{dz}>0$$

where,

(2) $$P\left(E=1|C_{d},H_{i}\right)=1/1+e^{-\left(\beta_{0}+\beta_{1}C_{d}H_{i}\right)}$$

and $F\left(C_{d}\cdot H_{i}\right)$ is a link function.

At a conceptual level, the expressions above relating to $H_{1}$ can be interpreted as follows:

The probability that consciousness will emerge—that is, the generative potential of consciousness—depends on the product of the system’s dynamic complexity ($C_{d}$) and its internal informational coherence ($H_{i}$). This relationship is defined as positive: the higher the value of $C_{d}\cdot H_{i}$, the more likely it is that the system possesses generative potential for consciousness. The proportional analogy between dF and dz is based on their differences. Consistent with the *analogia entis*, the emergence of generative potential requires proportionally sufficient discrepancies to produce a rupture within the system and trigger change. From this perspective, the question is not how closely dF and dz resemble each other, but rather how far apart they must be to create a proportionate difference capable of transforming the system, allowing something new to emerge that could give rise to consciousness.

## 6. Deriving generative consciousness

The first step in defining an equation that, through a ratio or quotient, can measure the degree of proportionality between two components—information and complexity—is to employ an empirically validated neurological schema (Aerts et al., 2016) that reproduces neural connections capable of generating conscious experience. One such validated schema is connectome 76 (Hagmann et al., 2008). Connectome 76 has been successfully replicated by independent laboratories (Honey et al., 2009), and its effectiveness in modeling conscious processes has also been applied clinically in the study of epilepsy (Jirsa et al., 2017).

In the following sections, we justify step by step the specification of the equations we propose and formally define a mathematical index designed to quantify the generative potential of consciousness. If the mathematical logic used to derive the proposed equation is grounded in empirical and neurological evidence about the circuits underpinning consciousness—and also aligns with the recommendations of biological naturalism as articulated by Anil Seth (2025)—then the central question becomes: what would happen if we emulated a system of integrated networks within AI that is capable of learning, dynamic plasticity, informational memory, processing, integration, and execution of sensory information—essentially, incorporating all the biological components observed in connectome 76 that contribute to generating conscious experience? In such a case, could our indicator be applied to assess proportional differential analogy and determine to what extent a system of this nature might support the emergence of conscious experience?

Three mathematical phases will define the development of this new index. The first phase involves the empirical grounding of the equations using connectome 76. The second phase will analyze simulations conducted within connectome 76 to examine the values and properties obtained when applying our equations to a circuit associated with resting-state consciousness. The final phase will extend our equations to a network system implemented in generative AI. Using this system, we will carry out large-scale simulations and analyze whether the results obtained in AI, when evaluated with our indicator, converge with those observed in connectome 76. If such convergence is demonstrated, we will have grounds to proceed with our proposed system of equations. The simulations employed the default connectome (76 regions) provided by *The Virtual Brain* (TVB), corresponding to a resting-state mental condition (Sanz-Leon et al., 2013). In each simulation run, the 15 brain regions exhibiting the lowest entropy in their neural mass activity were selected. The decision to select 15 out of 76 regions was based on the proposals of Tononi (2004, 2005) and the principles of IIT, from which perspective it has been suggested that relatively small systems can already emulate sensory information processing and integration, giving rise to conscious experience (Balduzzi and Tononi, 2008). Evidence presented by Oizumi et al. (2014), further demonstrated that between 8 and 12 nodes are necessary to reproduce IIT in intelligent systems, even in simplified implementations. These findings are consistent with other evidence aimed at computationally emulating neural circuits characteristic of conscious experience (Xie, 2025).

Although the primary measured variable was the neural activity in each region, conscious experience unfolds at a specific temporal instant. Therefore, the generative potential of artificial consciousness must be modeled as a time-dependent process. Let $x_{i}(t)$ denote the simulated signal at time t in region i, with n being the number of selected regions. By aggregating the signals from this informative subnetwork over time (in microseconds), one obtains a scalar approximation of the system’s collective dynamic behavior—a useful generative observer of the network’s internal structure.

Specifically, averaging n temporally coherent but spatially distributed signals suppresses local noise and enhances global patterns of coordination. Mathematically, this can be understood as a projection onto the constant vector basis $1\in R^{n}$, preserving common oscillatory components. The resulting function is Eq. (3):

(3) $$R(t)=\frac{1}{n}\sum_{i=1}^{n}x_{i}(t)$$

which is herein defined as the generative function of the system, capturing the emergent, time-resolved dynamics of the most informative cortical subnetwork. The next step is to formalize the underlying structure of the signal in a way that allows the estimation of its generative potential. To achieve this, we define two integral-based descriptors that capture distinct yet complementary aspects of the signal’s dynamic behavior: change and stability.

### 6.1. The first-order change

We begin by computing the absolute value of the first derivative of R(t), which reflects the rate of change of the signal—i.e., how fast and often it transitions from one state to another. This provides a proxy for informational activity or generative dynamism. The function is defined as shown in Eq. (4):

(4) $$\frac{dR(t)}{dt}=\frac{1}{n}\sum_{i=1}^{n}\frac{dx_{i}(t)}{dt}\Rightarrow \left|\frac{dR(t)}{dt}\right|=\left|\frac{1}{n}\sum_{i=1}^{n}\;\frac{dx_{i}(t)}{dt}\right|$$

To obtain a scalar descriptor, we integrate this quantity over the full-time interval [0,T] using Simpson’s rule (a numerical approximation to the definite integral), as shown in Eq. (5):

(5) $$\Phi =\int_{0}^{T}\left|\frac{dR(t)}{dt}\right|dt\approx Simpson\left(\left|\frac{dR(t)}{dt}\right|\right)$$

This quantity captures the informational flux of the generative system, representing how much activity the system produces over time.

### 6.2. The second-order change

To assess the stability (or smoothness*)* of this information flow, we compute the second derivative (see Eq. (6)):

(6) $$\frac{d^{2}R(t)}{dt^{2}}=\frac{1}{n}\sum_{i=1}^{n}\frac{d^{2}x_{i}(t)}{dt^{2}}\Rightarrow \left|\frac{d^{2}R(t)}{dt^{2}}\right|=\left|\frac{1}{n}\sum_{i=1}^{n}\;\frac{d^{2}x_{i}(t)}{dt^{2}}\right|$$

This derivative indicates the acceleration (or volatility) of the signal’s change—i.e., whether the system behaves erratically or in a regulated manner. Again, we integrate this magnitude across the full-time interval (see Eq. (7)):

(7) $$\kappa =\int_{0}^{T}\left|\frac{d^{2}R(t)}{dt^{2}}\right|dt\approx Simpson\left(\left|\frac{d^{2}R(t)}{dt^{2}}\right|\right)$$

Higher values of κ indicate increased dynamical irregularity, which approximates the complexity of the signal—a high κ suggests a less stable system with greater local entropy and unpredictability in its evolution. In this sense, κ functions as an estimator of dynamic complexity.

### 6.3. Defining the Attribution Consciousness Index (ACI)

The *Attribution Consciousness Index* (ACI) is defined as the ratio between generative activity and its second-order volatility (see Eq. (8)):

(8) $$ACI=\frac{\Phi }{\kappa }$$

This ratio expresses a trade-off between informational activity and dynamical irregularity (complexity). A higher ACI implies that the system is capable of producing rich activity patterns without excessive volatility, suggesting a more organized, potentially conscious-like structure of generativity.

### 6.4. Signal-range normalization

To ensure scale invariance and comparability across simulations, we normalize the ACI by the dynamic range of the signal (see Eqs. 9 and 10):

(9) $$R_{\text{range}}=max(R(t))-min(R(t))$$

and

(10) $${ACI}_{\text{norm}}=\frac{ACI}{R_{\text{range}}}$$

This step controls for amplitude-based biases, ensuring that the index reflects proportional structure rather than raw signal magnitude. In this sense, the formulation of the ACI coefficient shares a conceptual similarity with *Allan variance* (Stein, 2010). Both methods quantify how a signal changes when examined through time-windowed observations. In the case of Allan variance, one computes σ(τ) across different integration times τ, thereby assessing the signal’s stability as a function of temporal scale. Similarly, the ACI involves integrating over a fixed interval [0,T], with the resulting value inherently dependent on the scale of integration.

## 7. Interpretation of the coefficients and the ACI

**Fig. 1.**
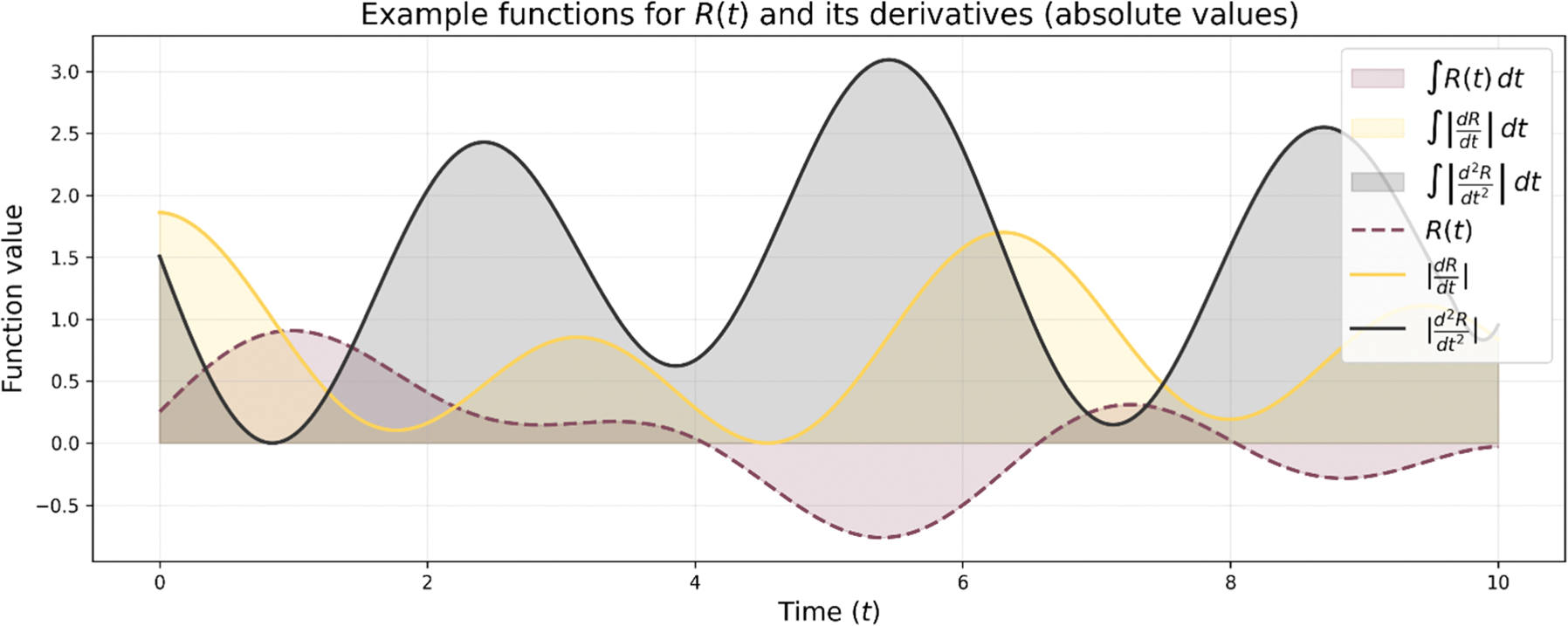
Pedagogical illustration of the areas computed by the two integrals that define the ACI coefficients Φ and κ.

To clarify precisely what the coefficients Φ and κ measure, Fig. 1 presents an example of a sinusoidal average function R(t), along with its first and second derivatives, and the areas computed by the corresponding integrals. As illustrated, the coefficient Φ captures the accumulated variability of the signal—that is, the informational richness of the system—while κ quantifies the area associated with the curvature of the second derivative. Higher values of κ indicate more irregular oscillations and reduced coherence, suggesting a more unstable and complex system with greater uncertainty.

More specifically, Φ quantifies the temporal variability of the average neural signal within a specific time window. It is interpreted as a measure of dynamic entropy or cumulative activity change. It is computed by integrating the absolute value of the first derivative of R(t)—the mean activity of the most informative subnetwork—over time (in milliseconds or microseconds). In contrast, κ estimates the dynamic curvature of the system, reflecting the variation in the rate of change of the signal. It corresponds to the second derivative of R(t) and measures how rapidly the signal’s complexity evolves over time.

The ratio ACI=Φ/κ defines the ACI, which expresses the proportional efficiency of information flow relative to the system’s rigidity or fluctuation speed. A higher ACI suggests an optimal functional balance between variability and control, interpreted here as a greater capacity for generating conscious states. This ratio can be normalized (see ${ACI}_{\text{norm}}$) to facilitate comparisons across different simulation runs and to yield a value expressed as a positive *odds ratio* (OR), which by definition holds $\forall_{OR}>0$.

## 8. Simulation-based analysis and results

### 8.1. Activation of brain structures during conscious experience

Using the TVB Connectome with 76 regions, we conducted 500 simulations, each representing a sensory process of conscious experience in the human brain during resting mental states. In these simulations, we measured the mean neuronal activity, its standard deviation, and entropy levels, and also computed the integrals Φ and κ, together with their ratio ACI and its normalization, as specified in Eqs. (9) and (10). In each simulation, the 15 nodes exhibiting the lowest entropy were selected, representing the structures with the highest degree of systematic, patterned functioning. Because it was not feasible to generate a neuroimage of all the structures chosen for every simulation, we opted instead to provide an anatomical and radiological summary indicating which areas were most frequently activated across the 500 simulations based on their low entropy.

**Fig. 2.**
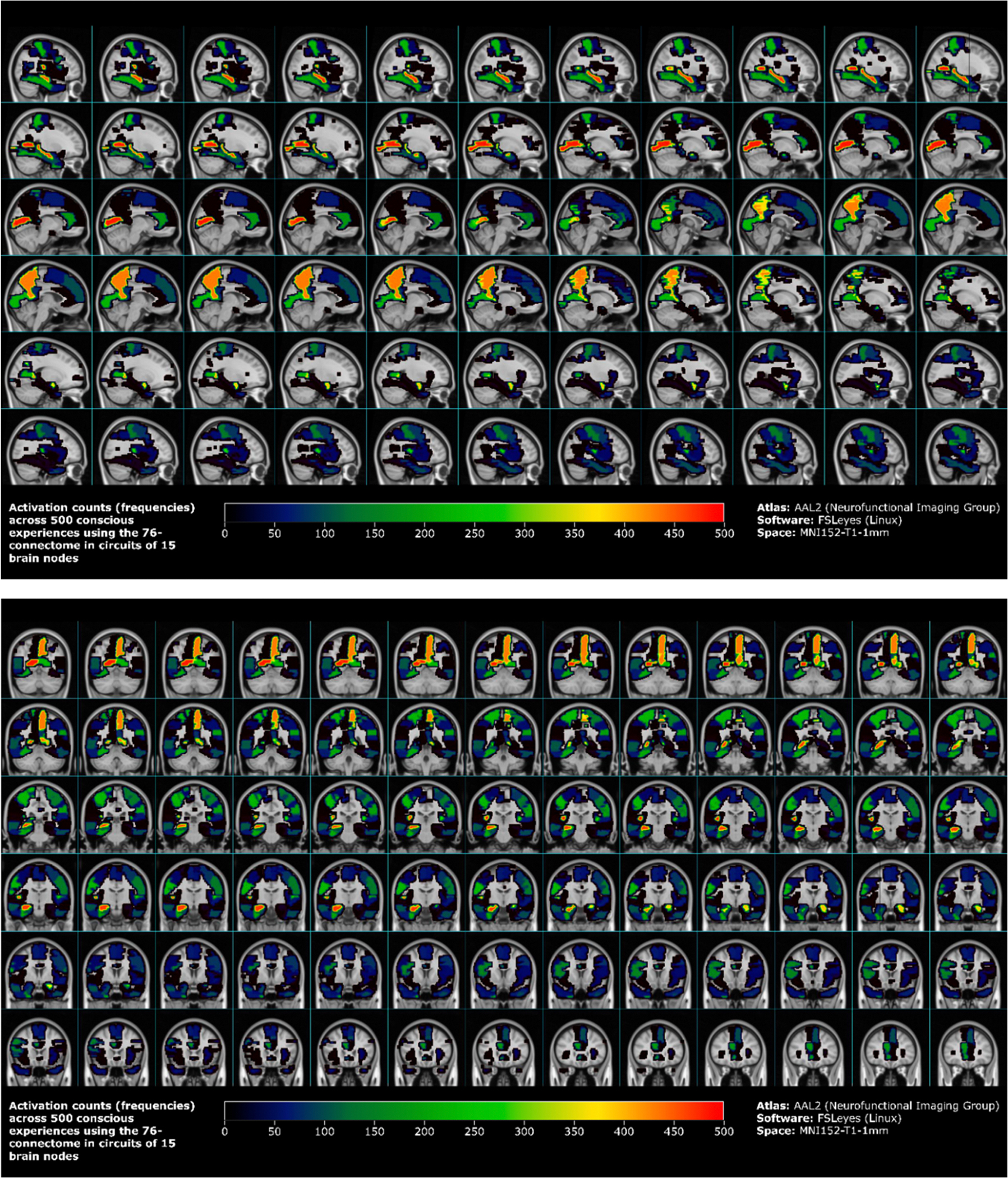

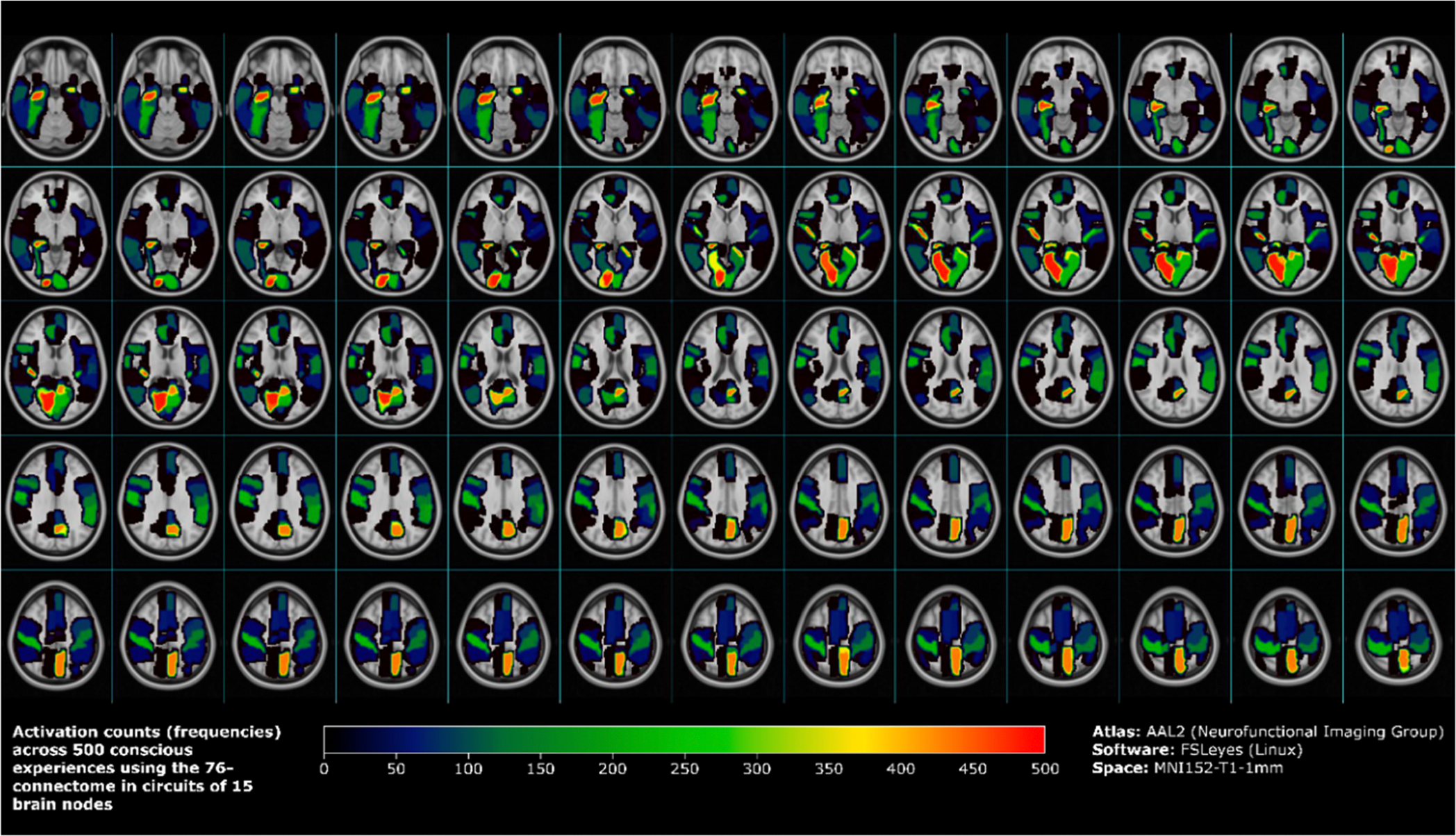
**A**. Sagittal MRI view in FSLeyes (Linux) showing 500 simulations of conscious experience using the validated connectome 76 (The Virtual Brain) with the AAL2 atlas and MNI152-T1–1mm space. **B**. Coronal MRI view in FSLeyes depicting 500 conscious experience simulations in connectome 76, based on the AAL2 atlas and MNI152-T1–1mm template. **C**. Axial MRI view in FSLeyes illustrating 500 simulations of conscious experience in connectome 76, using the AAL2 atlas and MNI152-T1–1mm space.

For each node, we recorded the frequency of selection across all simulations, resulting in frequency ranges between 0 and 500. If conscious sensory experience had been reproduced randomly across the 500 simulations, node frequencies would have displayed a uniform distribution. Such an outcome would be problematic, as it would suggest that despite the region-specific neuronal activity, there was no consistent signal pattern across the system, making it impossible to conclude that sufficient brain activations underpinning conscious experience had occurred. To present this information clearly, Fig. 2 shows a summary across three planes (sagittal, coronal, and axial) with 18 slices at different spatial *Magnetic Resonance Imaging (*MRI) levels. These visualizations were created using the AAL2 atlas (based on the anatomical spatial model ref. MNI152-T1–1mm) from the Neurofunctional Imaging Group and the FSLeyes software to generate MRI neuroimages. FSLeyes runs on Linux operating systems and is widely used in both clinical medicine and research for neuroimaging analysis. In this report, Fig. 2-A, Fig. 2-B and Fig. 2-C present only a summary with the most illustrative slices and views.

Figs. 2-A, 2-B and 2-C offer several compelling insights into the structures involved in conscious experience. The nodes showing the highest activation across the 500 simulations were lPFCDM, lCC, lHC, lAMYG, rPFCDM, rHC, rAMYG, and rA1. These nodes correspond to the following anatomical brain structures (listed in order): the dorsomedial prefrontal cortex (left hemisphere), the whole cingulate cortex (left hemisphere), the hippocampus (left hemisphere), the amygdala (left hemisphere), the dorsomedial prefrontal cortex (right hemisphere), the hippocampus (right hemisphere), the amygdala (right hemisphere), and the primary auditory cortex (Heschl’s gyrus, right hemisphere). The remaining nodes did not exhibit markedly elevated activation levels in the simulations. Moreover, the distribution of activation frequencies was not uniform, confirming that the 500 simulated conscious experiences were characterized by systematic functional patterns. The resulting distribution displayed a clear log-normal shape and structure.

These anatomical regions are consistent with previous scientific literature that has sought to identify the brain structures most critical in initiating conscious experience (Northoff and Ventura, 2025), as well as studies published in *Science* (Dehaene et al., 2017), and other work exploring correlations between these anatomical regions and the qualia of conscious experience (Chalmers, 1995). Based on these findings, we proceeded to analyze the statistical properties of the ACI and translated these structures into functional modules, enabling their reproduction in computational models of artificial intelligence.

### 8.2. Statistical properties of the ACI in brain activation patterns

At this stage of the results, we aim to analyze the statistical and mathematical behavior of the measurements obtained from the preceding 500 simulations. Table 1 presents the key descriptive statistics for all the variables examined. It also includes goodness-of-fit analyses using the Anderson–Darling and *Kolmogorov*–*Smirnov* (KS) tests to assess whether each variable followed a theoretical probability distribution suitable for modeling, prediction, and hypothesis testing.

Parameter estimation was conducted using the *Monte Carlo* method combined with Maximum Likelihood Estimation, with 1000 iterations. Once the parameters were estimated, we evaluated the extent to which each probability model could accurately represent our variables. In classical and Fisherian statistics, a KS *p*-value equal to or greater than 5 % is typically interpreted as evidence of a satisfactory fit. However, to visually validate the correspondence between the observed simulation data and the fitted models, it is also advisable to examine histograms overlaid with their respective density functions. For theoretical reasons, our analysis focused on the normal, log-normal, and gamma distributions.

**Fig. 3.**
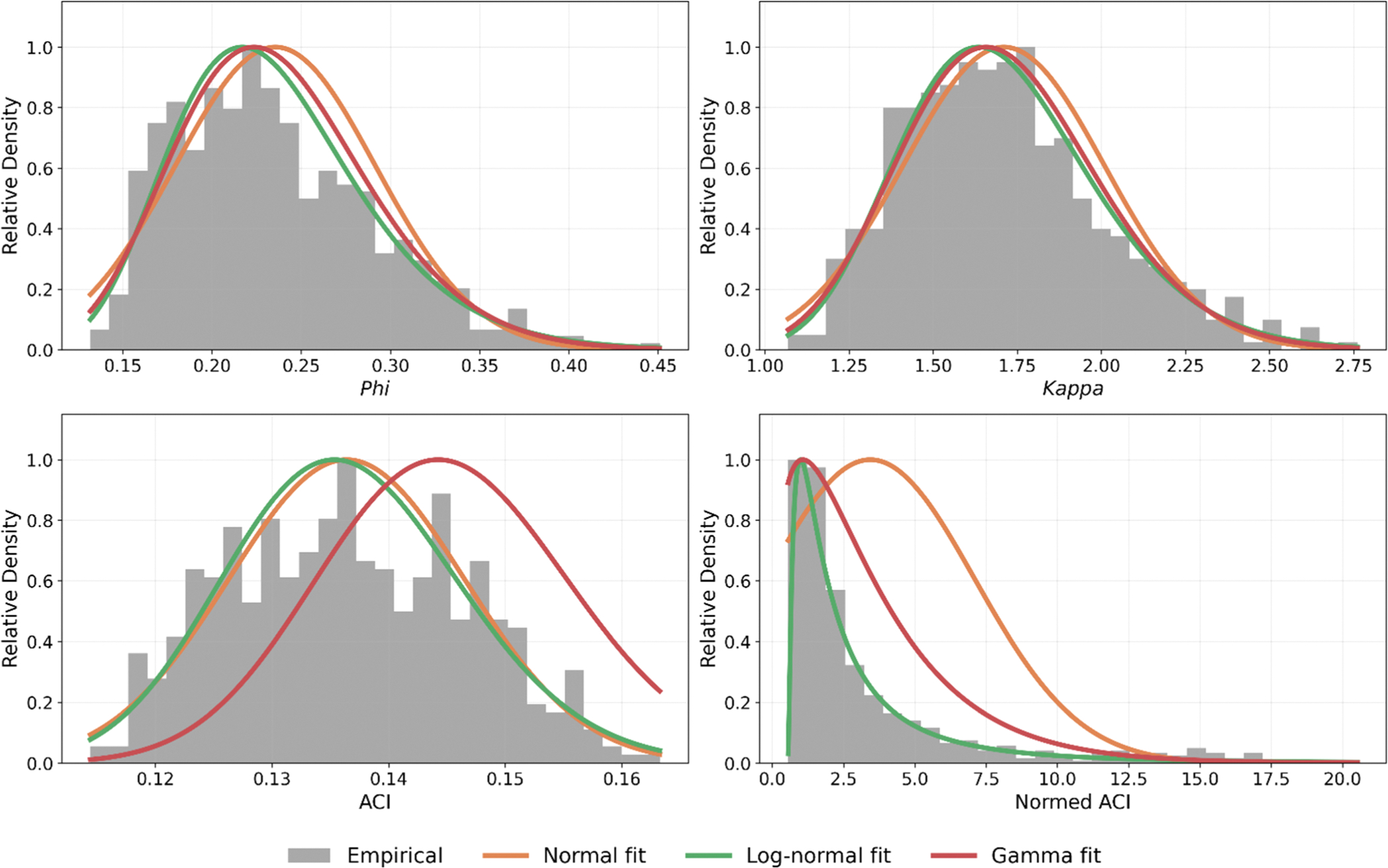
Histograms of the analyzed variables and probability models illustrate how the data observed across the 500 simulations—conducted using the empirical connectome 76, which enables the reproduction of conscious experiences during resting-state mental activity—can be predicted and modeled. Among the tested models, the log-normal distribution provided the best fit to the observed data. These visual findings are consistent with the KS goodness-of-fit tests, which yielded *p*-values exceeding 5 % for all variables.

Fig. 3 summarizes the distributions of the study’s key variables: the *Phi* (Φ) coefficient, *Kappa* (κ), ACI, and Normed ACI. Using the estimated parameters reported in Table 1, we plotted the probabilistic models to visualize how well they corresponded to the histograms of the observed data. Considering both the statistical results of the KS goodness-of-fit tests and the visual evidence provided in Fig. 3, we can conclude that the log-normal distribution was the most appropriate for modeling the observed data. This finding is especially relevant for the Normed ACI coefficient, which is expressed in OR metric and enables statistical interpretation and inference regarding the generative potential of consciousness across the 500 simulations performed using connectome 76.

It is essential to note that not every application or mode of using connectome 76 will necessarily give rise to conscious experience. The emergence of consciousness is not conceived—either within IIT or within Neo-Thomist logic—as a deterministic and univocal outcome, but rather as an analogical one. This consideration is precisely what compels us to adopt a stochastic framework for assessing the degree of certainty with which consciousness may occur.

The fact that the Normed ACI is expressed in OR metric offers a crucial advantage: its measurements can be transformed into percentages or probabilities, allowing us to quickly determine the extent to which the specific type of neuronal circuit activated by processing real sensory information (*D*) corresponds to the probability distribution that, in principle, should enable us to represent and model conscious experience (which, based on the initial results in Table 1 and the distributions in Fig. 3, is the log-normal distribution). This is expressed as a conditional probability, whereby the OR of the Normed ACI can be transformed into P(D∣Consciousness) using Eq. (11):

(11) $$P(D|Consciousness)=\frac{{ACI}_{\text{norm}}}{{ACI}_{\text{norm}}+1}=\frac{P(\text{Consciousness}|D)P(D)}{P(\text{Consciousness})}$$

From Eq. (11), we can derive Eq. (12), which provides a formal demonstration of why the Normed ACI, although expressed as an OR, can in fact be computed as a ratio of probabilities:

(12) $${ACI}_{\text{norm}}=\frac{P(\text{Consciousness}|D)}{P(\text{Consciousness}|\neg D)}$$

Of course, all of this is meaningful only if the log-normal distribution is indeed the one that allows us to represent conscious experience as an analogical product, expressible within the framework of the alternative hypothesis of this study: $P\left(E=1|C_{d}\cdot H_{i}\right),\;dF/dz>0$. This is the foundation that supports the Normed ACI and the core premise we aim to validate through the three phases described at the beginning of this subsection.

It is mathematically reasonable and indeed expected that an OR can be modeled using a log-normal distribution with pronounced right skewness. Suppose we have a neural circuit i and wish to determine with what level of certainty it has generative potential for consciousness. Using the Normed ACI, let us imagine that we obtain an OR of 18. Applying Eq. (11), we find that 18/19 = 0.947 = P(D∣Consciousness). The question we should pose in a hypothesis test is what the *p*-value would be, that is, $P\left(OR_{\text{N-ACI}}\geq OR_{\text{log-normal}}\right)$. Given that we are working with a log-normal model, the *p*-value can be calculated using the estimated parameter values 1.1108, 0.5327, and 1.5730, by integrating the area under the right tail of the curve. In this way, no further mystery remains. Recall that if X follows a log-normal distribution, the probability density function is shown in Eq. 13:

(13) $$f_{X}(x)=\frac{1}{x\sigma \sqrt{2\pi }}exp\left(-\frac{(lnx-\mu {)}^{2}}{2\sigma^{2}}\right)$$

The right-tail p-value is the integral from 18 to infinity (see Eq. (14)):

(14) $$p-value\;=\int_{18}^{\infty }\;f_{X}(x)dx$$

To compute this integral, we make the change of variable (see Eq. (15)):

(15) $$y=lnx,x=e^{y},dx=e^{y}dy$$

Substituting into the integral (see Eq. (16)):

(16) $$p-value=\int_{ln(18)}^{\infty }\;\frac{1}{e^{y}\sigma \sqrt{2\pi }}exp\left(-\frac{(y-\mu {)}^{2}}{2\sigma^{2}}\right)\left(e^{y}dy\right)$$

The $e^{y}$ terms cancel (see Eq. (17)):

(17) $$p-value=\int_{ln(18)}^{\infty }\;\frac{1}{\sigma \sqrt{2\pi }}exp\left(-\frac{(y-\mu {)}^{2}}{2\sigma^{2}}\right)dy$$

This is exactly the right tail of a normal distribution with mean μ and standard deviation σ (see Eq. (18)):

(18) $$p-value=\int_{ln(18)}^{\infty }\;\varphi (y;\mu ,\sigma )dy$$

where φ is the normal density. Finally, we define the Z-score (see Eq. (19)):

(19) $$z=\frac{y-\mu }{\sigma },dy=\sigma dz$$

Since (see Eq. (20):

(20) $$ln(18)\approx 2.890,z=\frac{2.890\;-\;1.1108}{0.5327}\approx 3.341$$

The p-value becomes (see Eq. (21)):

(21) $$p-value=\int_{3.341}^{\infty }\;\frac{1}{\sqrt{2\pi }}exp\left(-\frac{z^{2}}{2}\right)dz=1-\Phi (3.341)\approx 0.0004$$

This is the area under the log-normal curve to the right of 18. Therefore, with a Normed ACI of 18 in a brain circuit exhibiting generative potential for consciousness, the result 0.0004 < 0.001 leads to the rejection of the null hypothesis, stated as $P\left(E=1|C_{d},H_{i}\right)=P(E=\;1)$. It should be noted that in this case, the *p*-value is inversely proportional—though not exactly equivalent—to the probability P(D∣Consciousness). Specifically, 1−P(D∣Consciousness)=1−0.947=0.053, which, while not identical to 0.0004, tends to approximate the obtained p-value. This allows us to state that the higher the Normed ACI, the greater the likelihood that the observed circuit aligns with the distribution supporting the emergence of consciousness, a level of certainty expressed as P(D∣Consciousness).

However, what we have presented here is merely an illustrative example of the functionality and mathematical precision of the Normed ACI. The question we now pose—and which represents the central ambition of this study—is whether this same procedure could be applied to models of intelligent networks embedded in AI systems designed to emulate the biological and cerebral processes involved in conscious experience.

To explore this possibility, we must draw on the biological naturalism proposed by Anil Seth (2025) and emulate the organic processes occurring in the brain, thereby constructing networks that can learn, adapt, and modify themselves in response to specific types of stimuli. In this way, AI could integrate sensory information and ultimately trigger a decision or executive action. If this procedure proves functional in AI, we would be witnessing the development of the first mathematical coefficient capable of predicting the generative potential of consciousness. This is not an exaggeration but an acknowledgment of the significance of a scientific advance that would merit recognition, funding, and sustained research efforts in this direction.

### 8.3. Applying the ACI to AI neural architectures

The next phase aims to assess how ACI and Normed ACI behave within intelligent, plastic network systems designed to be integrated into generative AI.

#### 8.3.1. Specification criteria on AI architectures

One of the most challenging questions is how to define criteria that enable intelligent networks to emulate core brain processes underpinning conscious experience. While this example primarily illustrates how the ACI and Normed ACI coefficients can be applied in artificial systems, it captures only a narrow segment of their broader potential. To design a network that is both biologically plausible and fully configurable in Python, we incorporated the following key properties into its algorithms:

- **Hierarchical functional modularity:** the proposed model includes functional modules organized according to neuroanatomical levels, encompassing complex and multivalent systems such as visual, auditory, and somatosensory processing, integrative information functions, and executive capacities related to decision-making and adaptive learning. Incorporating this type of Python-based algorithm allows us to approximate, with a reasonable degree of reliability, the functional specialization of the human brain, which operates as a multimodal, outwardly directed network.
- **Connectivity characterized by dense, directed intra-module links and sparse inter-module links:** this connectivity logic is grounded in the principles of segregated efficiency and global integration, both of which are core features of brain organization as demonstrated in studies of neural networks (Bullmore and Sporns, 2009). If biological naturalism holds true, this characteristic represents one of the fundamental pillars necessary to emulate organic processes correlated with consciousness.
- **Nonlinear Hebbian plasticity:** this feature is critical because it emulates biological synaptic learning, wherein synaptic weights adjust dynamically in response to joint activation. The use of the tanh (pre × post) function provides saturation control and balances excitation and inhibition, thereby reproducing the more stable neural dynamics associated with conscious experience. Synaptic plasticity is regulated through the $W_{\text{max}}$ parameter, which in our system ranged from 1 (minimal plasticity) to 20 (maximal plasticity). These values are part of the Hebbian rule described in Eq. (22):
(22) $$W_{pn}(t+\Delta t)=clip\left[W_{pn}(t)+\eta tanh\left(x_{p}(t)x_{n}(t)\right),W_{min},W_{max}\right]$$
  Eq. (22) is combined with η, a parameter ranging from 0 to 1.1, which determines the system’s level of adaptive learning in response to its environment. In other words, η enables the system to learn recurrent activation patterns through local associations, a mechanism central to unsupervised learning processes. By setting different levels of learning capacity and permissible plastic changes in the network configuration, we can analyze how the accumulated information (Φ coefficient) and the processed sensory signal vary, thereby generating scenarios with varying degrees of complexity. These scenarios are evaluated using the integral or the κ curvature coefficient.
  In this way, we can explore potential relationships among κ,Φ,ACI, and Normed ACI with respect to specific characteristics of the proposed network model. To introduce sufficient variation in sensory signal transmission, we distributed $W_{\text{max}}$ values from 5 to 20 for each η value, organized in increments of 0.10, with 0.4 as the lowest and 1.1 as the highest. This procedure resulted in a minimum of 1921 simulations.
- **Differentiated sensory stimulation:** each node receives specific signals modulated by (1) pink noise (characteristic of and present in human brain electroencephalography during resting and conscious mental states) and (2) chaotic signals that emulate the real variability of the sensory environment and the functional resonance of regions involved in processes such as memory and attention.

#### 8.3.2. Why our proposed network can be trainable with AI

A key question is what enables this network to learn and adapt within generative AI systems. Its main foundation is the rule implementing Hebbian learning (Eq. 21), but three additional capabilities are critical. First, parameterized trainability: the hyperparameters η (learning rate) and $W_{\text{max}}$ (plasticity limits) make the system tunable to diverse environments, supporting a form of structural meta-learning. This feature could allow the network not only to detect patterns but also to recognize how those patterns improve adaptive responses to changing demands. While this remains to be validated computationally, it is mathematically plausible within the proposed architecture. Second, generalization capacity emerges from combining structured randomness in sensory inputs with differentiated resonances, fostering plastic adaptation and self-organization. Learning goes beyond simple reinforcement, engaging deeper processes of information integration and meaning attribution. The network develops internal representations capable of anticipating regularities, inferring causal relationships, and autonomously reorganizing its functional architecture. This synthesis of plasticity and resonance guides experience toward coherent configurations and underpins its potential for emergent intelligence. Third, the model avoids absolute determinism by introducing noise and chaotic signals that sustain entropy and induce nonlinear dynamics (Izhikevich, 2007). These dynamics emulate core properties of complex adaptive systems like the brain, supporting the generative potential for consciousness.

#### 8.3.3. Formal definition and functional structure of the network

Our network comprised eight functional modules distributed across 27 nodes, designed to emulate specific brain functions involved in conscious experience. The modules were distributed as follows: four nodes were *Visual* (V), three were *Auditory* (A), three were *Somatosensory* (S), four were dedicated to *Information Integration* (I), four supported *Executive Functions* (EX), three emulated *Memory Functions* (M), three performed *Attentional Functions* (AT), and the remaining three were associated with *Salience Levels* (SA).

Among these, the functional modules with the highest number of connections were those responsible for I, which maintained extensive links with all other modules. Mathematically, the formal expression describing the node activation dynamics is presented in Eq. (23):

(23) $$x_{n}(t)=tanh[\underset{\text{Sensory}\;\text{input}}{\underbrace{g_{n}s_{n}(t)}}+\underset{\text{Global}\;\text{inhibition}}{\underbrace{\lambda x_{n}(t-\Delta t)}}+\sum_{p}W_{pn}(t)tanh\left(x_{p}(t-\Delta t)\right)]$$

Activation dynamics refers to the temporal evolution (in our case, states up to 10 microseconds) of each node as a function of (a) the differentiated sensory signal, (b) global inhibition, and (c) the sum of presynaptic inputs modulated by dynamic weights. Accordingly, the terms in Eq. (23) are defined as follows: $g_{n}$ = individual sensory gain factor; λ<0 = global inhibition factor; and $s_{n}(t)=$ sensory input signal of the node. The remaining terms were introduced and explained in the nonlinear modular Eq. (22).

To objectively summarize the structural functionality of our network, we report here the results of the primary indicators describing the model’s properties. Across the 1921 executions, the network consistently maintained the same levels of signal density (i.e., the proportion of connections among nodes (Boccaletti et al., 2006), which in our system was 0.3647), clustering (the distances and local grouping among nodes (Watts and Strogatz, 1998), which was 0.5556), efficiency (the amount of sensory signal processed (Latora and Marchiori, 2001), which reached 0.7009), and average path length, which measures the extent to which the architecture supports information integration (Barabási, 2016).

**Fig. 4.**
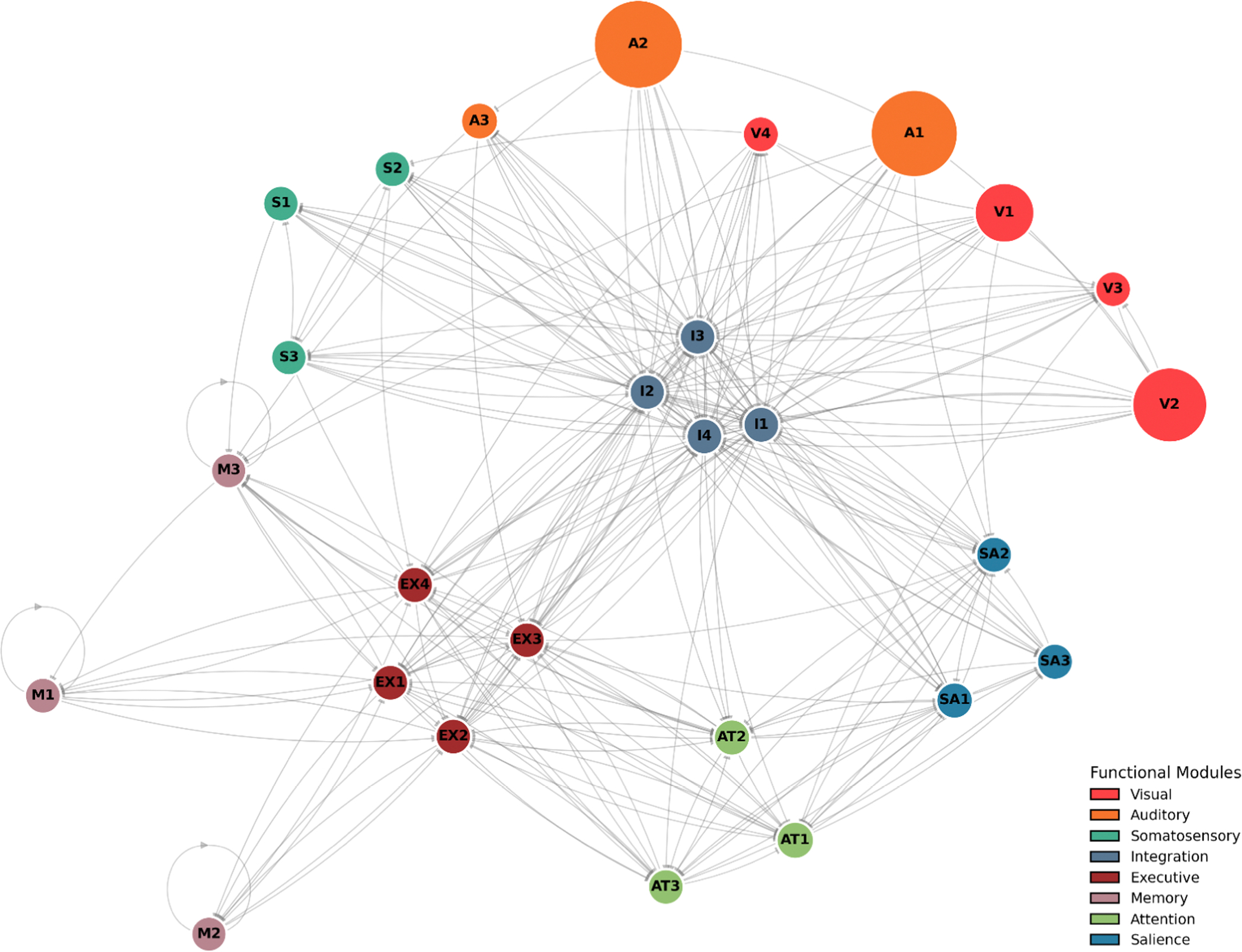
Computational network model designed to emulate organic processes involved in processing sensory signals, integrating them, and potentially supporting executive functions for decision-making. Structural properties of the network: density = 0.3647, clustering coefficient = 0.5556, global information efficiency = 0.7009, and average path length = 1.7654. Functional modules: V= visual, A= auditory, S= somatosensory, I= integration, EX= executive, M= memory, AT= attention, and SA= salience.

This measure is based on the concept of “hops”: the more hops information requires to travel between nodes, the greater the informational dispersion. Because we aimed to emulate biological and neural processes that reproduce the connectivity patterns observed in conscious experience, it was important for this value to remain close to one. In our configuration, the average path length was 1.7654 across all simulations, indicating that the system facilitates integration and, consequently, according to the principles of IIT, would also support the emergence of conscious experience (assuming it arises via the logic of proportional analog differences, as posited in our hypothesis). Fig. 4 shows the composition and graph representation of our network.

**Fig. 5.**
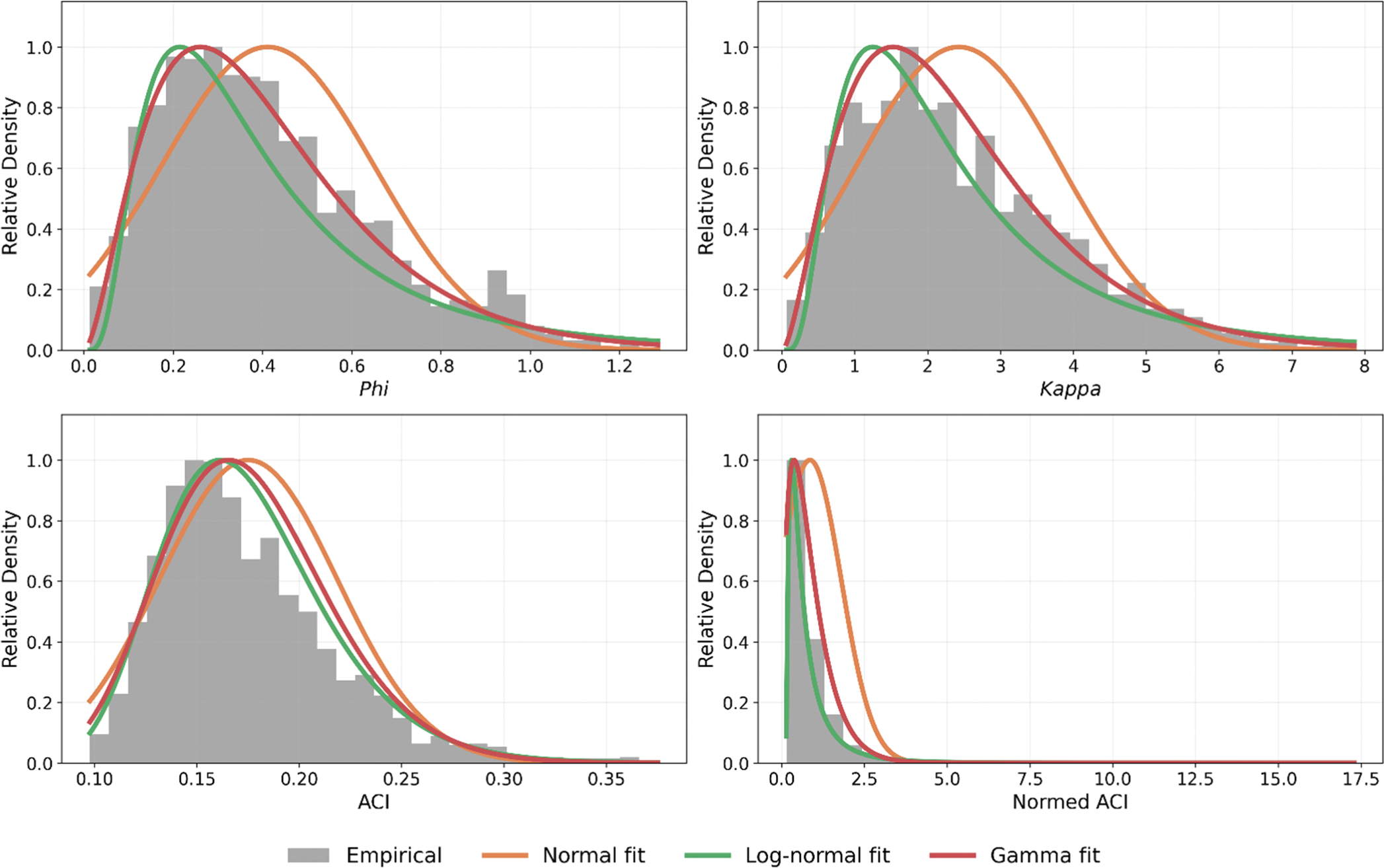
Distributions of the observed data, processed by our intelligent network model, were examined across 1921 executions alongside theoretical log-normal, gamma, and normal distributions to assess the goodness of fit visually. The parameters of the theoretical distributions were estimated using *Monte Carlo* methods with maximum likelihood over 1000 iterations. Goodness-of-fit tests indicated that the Normed ACI adheres to a log-normal distribution, supporting its suitability for predictive modeling.

Although these properties remained constant across all 1921 simulations, the entropy levels (Shannon, 1948) of the nodes and the variability of signal activity (Deco and Kringelbach, 2016) changed with each execution, thereby producing signal patterns that were similar but not identical. They were similar because the structural basis of the network remained fixed, but the $W_{\text{max}}$ coefficients (reflecting plastic changes) and η (the network’s learning capacity) varied, as did the ACI coefficients. Using the *Monte Carlo* method with maximum likelihood estimation over 1000 iterations, we estimated the parameters of log-normal, gamma, and normal distribution models to determine whether the ACI values and the terms in their equations followed any consistent pattern. Fig. 5 presents the distributions of the terms corresponding to the ACI and Normed ACI equations.

**Fig. 6.**
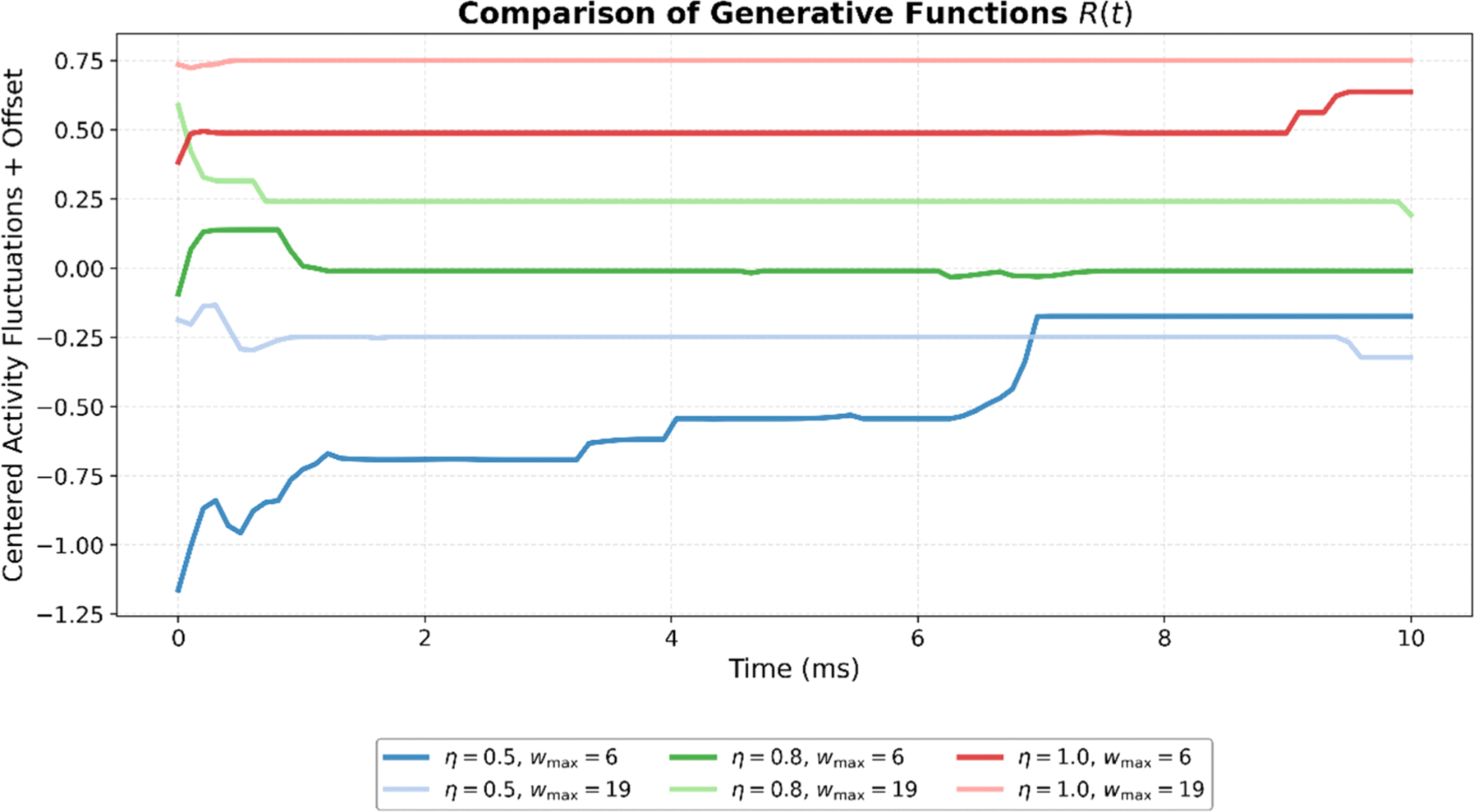
Scaled visualization of the R(t) functions using different hyperparameter conditions in the Hebbian rule. Three η levels are considered (low, medium, and high). For each η level, we provide two $W_{\text{max}}$ values: one low and one high. This approach allows us to examine how hyperparameter variability could affect the signal.

In the case of fitting the intelligent network with eta parameters ranging between 0.4 and 1.1, representing the adaptive learning capacity of the artificial model, and plasticity levels spanning from 1 to 20 for each eta learning level, the estimation yielded the following results for the four target variables: for *Phi*, the normal distribution parameters were μ=0.4118 and σ=0.2391 (Anderson-Darling statistic = 26.5460, KS *p*-value < 0.0001), the log-normal distribution parameters were shape = 0.6760, loc = 0.0000, and scale = 0.3390 (KS *p*-value < 0.0001), and the gamma distribution parameters were shape = 2.7294, loc = 0.0000, and scale = 0.1510 (KS *p*-value = 0.7764). For *Kappa*, the normal distribution parameters were μ = 2.4273 and σ = 1.4106 (Anderson-Darling statistic = 24.0287, KS *p*-value < 0.0001), the log-normal parameters were shape = 0.6841, loc = 0.0000, and scale = 1.9917 (KS *p*-value < 0.0001), and the gamma parameters were shape = 2.6850, loc = 0.0000, and scale = 0.9047 (KS *p*-value = 0.1767). For ACI, the normal distribution parameters were μ = 0.1751 and σ = 0.0435 (Anderson-Darling statistic = 29.7168, KS *p*-value < 0.0001), the log-normal parameters were shape = 0.2329, loc = 0.0000, and scale = 0.1702 (KS *p*-value = 0.0011), and the gamma parameters were shape = 18.0022, loc = 0.0000, and scale = 0.0097 (KS *p*-value < 0.0001). Finally, for Normed ACI, the normal distribution parameters were μ = 0.8565 and σ = 0.9450 (Anderson-Darling statistic = 175.8429, KS *p*-value < 0.0001), the log-normal parameters were shape = 0.9692, loc = 0.1229, and scale = 0.4609 (KS *p*-value = 0.4888), and the gamma parameters were shape = 1.7899, loc = 0.0000, and scale = 0.4799 (KS *p*-value < 0.0001). Based on these results, we conclude that the Normed ACI follows the statistical law of the log-normal distribution, which is a relevant finding for enabling predictions and hypothesis testing using this indicator. Fig. 6 shows the R(t) functions over a state of up to 10 microseconds processed in our network model for different values of the hyperparameters η and $W_{\text{max}}$.

#### 8.3.4. Correlations between Hebbian learning, entropy, and Normed ACI

Because the primary indicator of interest is Normed ACI, we sought to determine whether its values were related to the functional properties of the modular network we designed (see Fig. 4). Analyzing this potential relationship would be computationally valuable for two reasons.

First, it would help clarify whether the emergent perspective on consciousness—within the framework of biological naturalism—might be influenced not only by the ratio between the amount of information and the level of complexity (curvature) but also by organic learning processes and potential plastic changes. Second, such an analysis would have practical utility by enabling us to refine and improve our network model to produce a more accurate distribution of Normed ACI, which is more sensitive to detecting the generative potential of artificial consciousness.

**Fig. 7.**
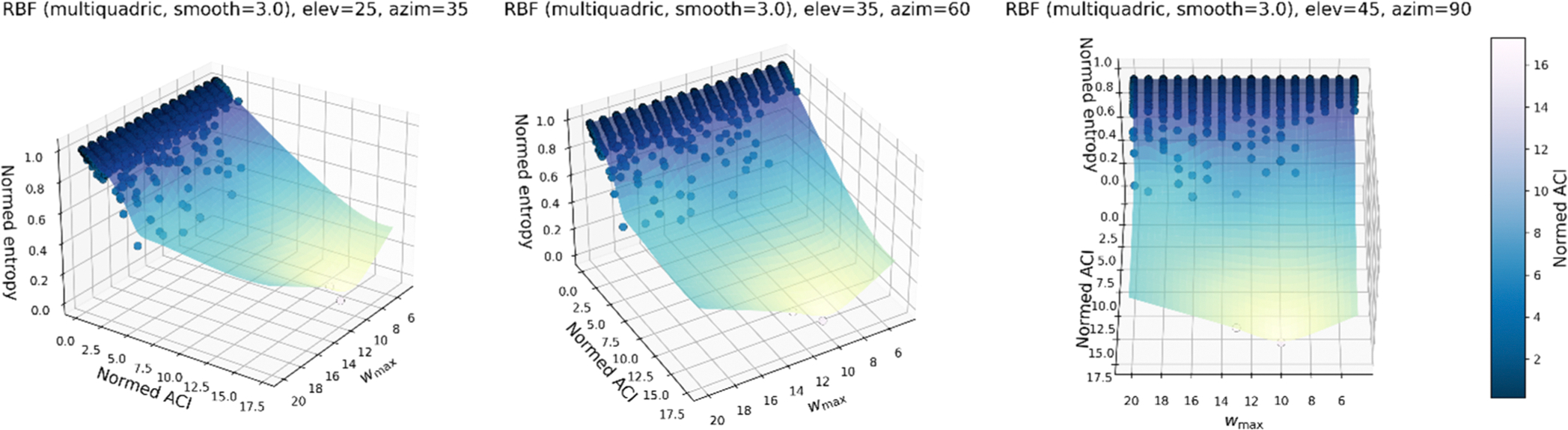
3D correlation visualization using multiquadric *Radial Basis Function* (RBF) interpolation between the variables $W_{\text{max}}$, standardized Shannon entropy levels, and Normed ACI. A clear decreasing trend is observed across all $W_{\text{max}}$ levels between entropy and Normed ACI. This indicates that as entropy decreases—and thus the signal and neuronal dynamics per node become more systematic—the Normed ACI tends to increase. The terms “elev” and “azim” refer to the parameters used to adjust the planes and rotations of each plot.

To explore potential associations, we computed the *Mutual Information Index* (MII) among entropy, $W_{\text{max}}$, and Normed ACI across the 1921 executions of our model. Specifically, the MII between Normed ACI and entropy was 1.1898, while the MII relative to $W_{\text{max}}$ was 0.0425. For all other variables, the MII was close to zero. These results suggest that Normed ACI and entropy share information that is not necessarily linear but provides an initial indication of which variables warrant further correlation analysis. Accordingly, we computed a three-dimensional correlation using a *Radial Basis Function* (RBF) multiquadric kernel interpolation, whose function is defined in Eq. (24):

(24) $$Z(x,y)=\sum_{i}\lambda_{i}\sqrt{{\left(x-x_{i}\right)}^{2}+{\left(y-y_{i}\right)}^{2}+\epsilon^{2}}$$

where ϵ=3. This procedure yields a smooth approximation of the functional relationship among the variables, facilitating a visual exploration of their correlations. Fig. 7 shows the visualizations of the 3D correlations using RBF.

The results and trends presented in Fig. 7indicate a clear relationship between the entropy levels of the dynamic signal activity processed by the nodes and Normed ACI. Specifically, as entropy decreases—producing a more systematic signal—Normed ACI tends to increase. This pattern corresponds to a negative linear correlation that aligns precisely with the logic observed in connectome 76: the structures or nodes that most effectively support ACI are those exhibiting the lowest entropy levels. The optimal range of plasticity ($W_{\text{max}}$) for achieving Normed ACI values greater than 10 units lies between 10 and 14. This observation is particularly noteworthy, as it suggests that the highest probabilities of consciousness emerging within an AI system would likely require $W_{\text{max}}$ scores in the range of 10–14, as per the Hebbian learning rule.

These findings further suggest that Normed ACI provides a mathematical basis for validating the Φ coefficient as a meaningful measure. Importantly, our Φ does not quantify levels of integration per se, but rather the *amount of informational flow within the system*—that is, network-level dynamics reflecting the potential information the system may be capable of integrating over time. While conceptually related to the Φ coefficient in IIT, it is not equivalent. Accordingly, as Φ increases and κ decreases, the overall value of Normed ACI should rise. When the available information for integration (Φ) is high and κ—representing dynamic irregularities—is low, entropy is expected to decline as ACI increases.

Taken together, these findings support not only the internal logic and mathematical structure of the ACI formulation, but also its broader theoretical coherence within the context of consciousness modeling.

### 8.4. Empirical validity of the Normed ACI

Although the Normed ACI coefficients derived from both connectome 76 and our network model exhibit certain statistical similarities (particularly, in both cases their values appear to follow a theoretical log-normal distribution) two forms of validity must be assessed. The first concerns the *empirical validity* of their distributions. For other researchers to adopt Normed ACI as an inference tool to quantify the generative potential of consciousness, it is not sufficient merely to establish statistical modeling rules. Nor is it enough that the modular processes of our computational network resemble the biological architecture underlying conscious experience. It is essential to mathematically demonstrate that the values produced in simulations empirically converge and align with the actual values.

Specifically, the empirical validity presented here entails demonstrating mathematically that the observed distribution of Normed ACI values from connectome 76 empirically matches the values generated by executing the same index in our modular AI network. To achieve this, and to remain as faithful as possible to the observed distributions of our variables—Normed ACI AI (the index produced by our network) and Normed ACI Human (the index derived from connectome 76)—we applied *Kernel Density Estimation* (KDE). This approach enables the estimation of a function that best captures the true underlying distribution of the data.

At this stage, relying solely on log-normal parameters would be insufficient, as this procedure would model Normed ACI against a known statistical distribution. The distinction here is critical: the objective is to assess how well the observed empirical values—those potentially present in the human brain—align with the Normed ACI values produced by a computational AI model designed to emulate the organic processes of consciousness. Applying KDE yielded functions that could be directly compared to determine what proportion of the area under the curves was shared between the biological (human) data and the AI-generated data. The most precise metric for this purpose is the *Overlap Coefficient* (OVL) (Anderson et al., 2012), complemented by two additional measures: *Jensen*–*Shannon Divergence* (JSD) (Endres and Schindelin, 2003) and Hellinger Distance (Pollard, 2001). While OVL quantifies the exact proportion of overlapping area under the curves—indicating the degree of alignment between the two distributions—JSD assesses divergence in their shapes, and Hellinger Distance measures non-identity based on the distance between functions.

**Fig. 8.**
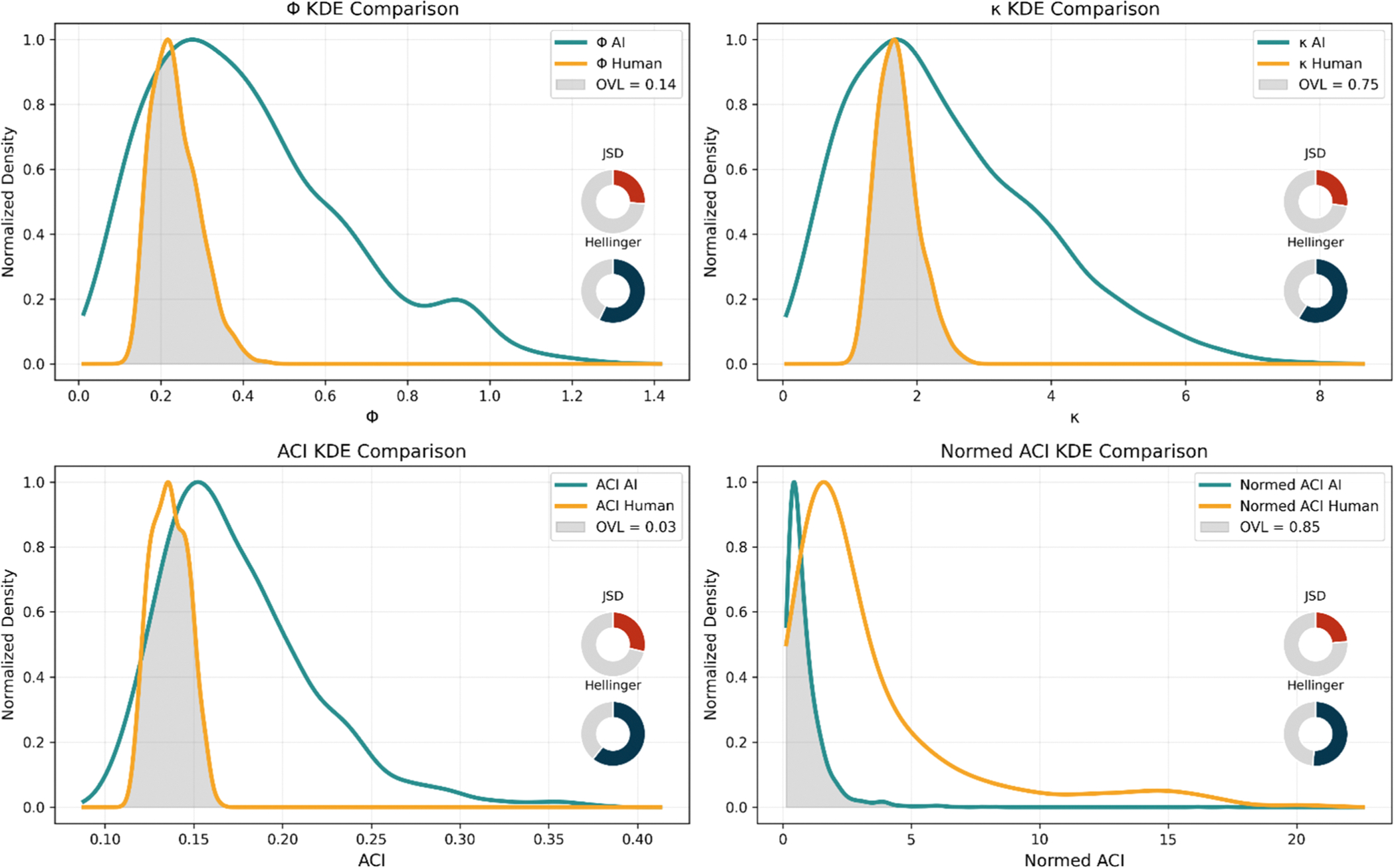
Application of *Kernel Density Estimation* (KDE) and analysis of the similarities between the terms of the Normed ACI integrals produced by AI and those generated by a real human brain. For these measurements, the *Overlap Coefficient* (OVL, threshold >0.7), *Jensen*–*Shannon Divergence* (JSD, threshold <0.3), and Hellinger Distance (threshold ~0.5) were used.

To establish empirical validity between functions, it is generally recommended that OVL exceeds 0.7 (Anderson et al., 2012), JSD remains below 0.3 (Endres and Schindelin, 2003), and Hellinger Distance approximates 0.5 (Pollard, 2001). These results are presented in Fig. 8, which mathematically demonstrates that the Normed ACI produced by the AI achieves an empirical correspondence with the human Normed ACI of approximately 85 %.

### 8.5. Predictive validity of the Normed ACI

The second type of validity is *predictive validity*. While in the previous subsection we demonstrated mathematically and statistically that an AI system can reproduce organic processes that are statistically functional for emulating conscious experience, our objective here is to quantify the extent to which Normed ACI measurements derived from an AI circuit can predict the neuronal circuits underlying conscious experience. We do not consider it sufficient merely to establish empirical similarities between density functions; rather, we also aim to quantify whether there is genuine predictive value. Although this might appear ambitious, having access to the Normed ACI values, connectome 76 data, the AI model, and fully controlled algorithms allows us to estimate the capacity of our neural and computational framework to predict the organic processes implicated in consciousness.

Because the Normed ACI values for AI and Human do not share the same observational units—in other words, each row contains independent values in each column—there is no basis for applying classical linear prediction. Instead, we adopted an analytical strategy widely used in the training architectures of generative AI systems.

The first step involved establishing associations through statistical matching. As we demonstrated in subsection 8.3.4, entropy levels share essential information with any expression of Normed ACI. Based on this evidence—which is both theoretically coherent and consistent with the two major theories of consciousness (IIT and GWS)—we decided to match Normed ACI AI values to Normed ACI Human values by aligning them according to their entropy levels. This matching served as the key criterion for training an intelligent model using *Kernel Ridge Regression* (KRR).

KRR is a form of analytical regression that relies on a nonlinear kernel function, which performs a grid search across the hyperparameters alpha, gamma, and the kernel itself (commonly *a Radial Basis Function*, RBF). Specifically, a grid search is an algorithm that statistically identifies the optimal combination of these hyperparameters by defining a set of possible values for each parameter (the “grid”). The model is then trained on each grid configuration, and performance is evaluated through cross-validation. Finally, KRR selects the combination that yields the best results and extracts prediction fit metrics.

In our case, we were particularly interested in assessing whether any combination of grid parameters across the Normed ACI AI and Normed ACI Human values would allow training a model capable of establishing predictive value. This predictive capacity would specifically target Normed ACI Human, indicating that we might not only have a generative potential for consciousness that is statistically modellable—as shown in Section 8.4—but also the ability to predict conscious experience through organic processes emulated by AI.

It is important to clarify that KRR is a supervised, nonlinear machine learning procedure designed to detect patterns. The intelligent model learns to identify these patterns and make predictions based on them. Because the prediction process is nonlinear, the resulting correlation coefficients, explained variance, and other fit metrics require adjustments that we will detail in the results section. What is essential here is that the reader understands the rationale underpinning the analytical procedure we applied. At the mathematical level, in our analyses, the formal equation defining our predictions was Eq. (25):

(25) $$\hat{y}(x)=\sum_{j=1}^{n_{\text{train}}}\alpha_{j}\times exp\left(-\gamma {\left\|x-X_{\text{train}}[j]\right\|}^{2}\right)$$

Eq. (26) is the function employed in the KRR:

(26) $$\hat{y}(x)=\sum_{i=1}^{n}\alpha_{i}K\left(x,x_{i}\right)$$

where and γ=0.1, and $\alpha_{i}$ coefficients are learned during the training process.

**Fig. 9.**
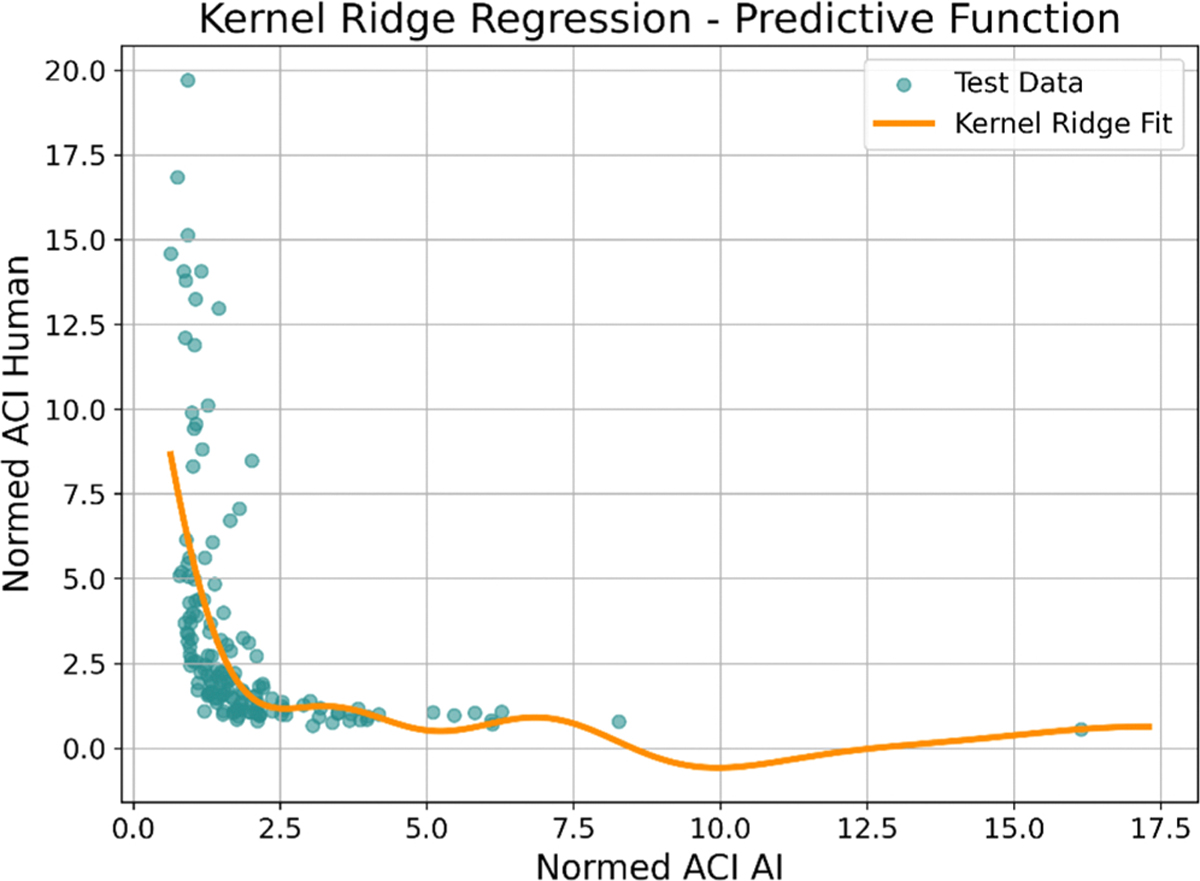
Kernel Ridge Regression predictive function modeling the relationship between Normed ACI AI and Normed ACI Human scores. The scatter plot displays the observed test data points, while the orange curve represents the nonlinear function learned by the model to detect systematic patterns. This function predicts Normed ACI Human values based on Normed ACI AI inputs, capturing both the strong inverse association in the lower range and more subtle variations at higher values.

The results indicated that the KRR was able to generate systematic nonlinear learning by detecting patterns that predicted 38.4 % of the variance in Normed ACI Human, with an RMSE (*Root Mean Squared Error*) = 2.926 and an MAE (*Mean Absolute Error*) = 1.796, a mean relative error of 0.523, a bias of –0.228, and a Spearman correlation of 0.807. The optimal hyperparameters were alpha = 0.01, gamma = 0.1, and kernel = radial basis function. Fig. 9 displays the scatter plot and the visualization of the learned function for pattern detection.

The results of the KRR analyses regarding the predictive validity of Normed ACI support the conclusion that AI, using our foundational computational model and the equations that define ACI, can successfully predict the Normed ACI Human measurements associated with neuronal circuits engaged during conscious experience. This finding allows us to assert—at least partially and from a rigorous statistical perspective—that the generative potential of AI is indeed measurable through Neo-Thomistic logical and mathematical principles. Consequently, we are able to reject our null hypothesis, thereby lending support to the probabilistic foundation of the *analogia entis*, which posits that the likelihood of consciousness emerging depends on the product of a system’s dynamic complexity and its internal informational coherence. This relationship is positive and is quantified through the Normed ACI: the higher this coefficient, the greater the generative potential for consciousness. In the Discussion (Section 9), we will examine specific threshold values of Normed ACI and propose statistical hypothesis tests that enable other independent researchers to apply Normed ACI in their AI systems, making robust, well-founded inferences and generalizations.

## 9. Discussion

### 9.1. Statistical criteria for generalization and Normed ACI thresholds

The formulations and data presented here could prove valuable for several types of statistical generalizations in neuroscience. From a clinical and neurological perspective, the most important pertains to estimating the parameters that allowed us to derive the theoretical log-normal distribution for Normed ACI in the 500 brain simulations using connectome 76—a connectome that has been empirically validated across thousands of patients from diverse countries and clinical profiles. These parameters were: 1.1108, 0.5327, and 1.5730. Using the equations and derivations provided in Section 8.2 (Eqs. (13) through (21)), it becomes feasible to conduct a basic hypothesis test (with a *p*-value) employing Normed ACI to analyze discrepancies between a patient’s neuronal activity—particularly when clinicians are uncertain whether the patient will regain self-awareness—and the theoretical Normed ACI distribution derived from connectome 76. To do so, one would record the patient’s observed data, compute the function R(t) (see Eq. 3), its integrals, the ACI ratio, and the normalized odds ratio (Normed ACI). With one Normed ACI value per patient and pre-specified theoretical thresholds corresponding to different significance levels, it is then possible to derive a *p*-value for the area under the curve, as demonstrated in Eqs. (13) through (21).

If the null hypothesis—asserting that the patient’s brain cannot generate self-consciousness—were rejected (in the strictest statistical interpretation, this would require Normed ${ACI}_{\text{patient}}>19$), then even in cases of coma where prognostic uncertainty is maximal, Normed ACI could serve as an evidence-based tool to predict the potential recovery of conscious experience. The ethical and legal implications of such predictions could be transformative for healthcare systems as we know them. For example, consider a coma patient whose brain activity yields a Normed ACI exceeding 19 (which corresponds to *p*-value < 0.05). Even if the patient’s clinical condition is poor and the prognosis negative, the mere fact that Normed ACI indicates the brain retains generative potential for consciousness would lend support to those advocating for maintaining life support. Importantly, Normed ACI would not be signaling that consciousness is present, but rather that, according to statistical prediction with a *p*-value, the patient’s brain could eventually regain self-awareness. If this possibility were empirically demonstrable—and it is, using Normed ACI—it raises profound questions: how many patients might not have been disconnected from intensive care simply because, at some point, they might have awakened?

While this example is deliberately striking, it is essential to emphasize that our proposal is merely speculative: a scenario designed to illustrate the future applicability of Normed ACI in real-world medical contexts, thereby provoking reflection on the ethical and moral dimensions of life-support decisions. Naturally, any scientist would agree that such an example remains conjectural and would require extensive empirical validation under rigorous medical and scientific standards before it could be systematically implemented. In this regard, we offer the potential of a new line of research that could transform and improve certain medical decisions currently entrusted to patients’ significant others—individuals who, without specialized knowledge, must decide whether to withdraw life support for a loved one in a coma. With Normed ACI, in situations where no advance directive exists, and to spare families the burden of such agonizing decisions, it would be possible to obtain an objective statistical estimate.

Recall that if Normed ACI is an odds ratio, then by definition, any Normed ACI value above 10 constitutes a statistical basis for rejecting the null hypothesis of no generative potential for consciousness. Accordingly, this threshold implies a high degree of certainty that the patient will regain awareness (10/10 + 1 = 0.91 × 100 = 91 % probability). While this reasoning applies to any statistical generalization based on hypothesis testing with Normed ACI, it is also true that, in the clinical example presented here, AI itself has not yet been incorporated. Up to this point, the generalization and inference rely exclusively on comparing the theoretical Normed ACI distribution estimated in this study with the observed Normed ACI derived from a patient’s neuronal activity by integrating the *R*(*t*) derivative functions and computing the observed Normed ACI for the patient.

Even this discussion alone would already justify a separate scientific report devoted exclusively to assessing how ACI could be applied in clinical practice. However, the fact that Normed ACI has also been implemented within an intelligent computational model replicating the brain’s organic architecture (and validated against connectome 76) opens the possibility of a second form of generalization: predicting which AI systems, analogous to the coma patient scenario, could be capable of developing conscious experience.

If researchers wish to apply the same logic of hypothesis testing, they could alternatively use the parameters from our computational model to define the theoretical log-normal distribution. In this case, the estimated parameters were: shape = 0.9692, loc = 0.1229, and scale = 0.4609. The procedure would then be as follows: in a generative AI system implementing our computational model (or improved versions), properly trained and deployed for human interaction (as is the case with ChatGPT, for example), it would be possible to calculate the observed Normed ACI. One could then use the theoretical log-normal distribution with these parameters, compute the integrals (i.e., the area under the *p*-value curve), and make a probabilistic, evidence-based decision.

In this context, the *p*-value would be interpreted as the probability that the theoretical Normed ACI is equal to or exceeds the observed Normed ACI. If the *p*-value were very small, for example < 0.01, this would mean that, out of 100 attempts to predict the generative potential for consciousness, at least one would be incorrect, implying 99 correct predictions. Such a result would provide a highly robust source of evidence supporting the likelihood that a generative AI (under the specified conditions) could develop consciousness.

To our knowledge, this is the first time in the indexed scientific literature that a proposal has been made to predict consciousness in generative AI systems using evidence-based methodologies rather than purely theoretical deductions, speculation, or philosophical argument. We strongly encourage other researchers—whether clinicians or not—to build upon the hypothesis testing framework proposed here (both for patients in intensive care and for generative AI) to refine, adapt, or advance the mathematical foundations and results derived from this study. We acknowledge that proposing the use of Normed ACI in clinical and AI contexts is, at this stage, premature and speculative. Nevertheless, speculation does not preclude rational inspiration, nor does it prevent us from suggesting which statistical analyses should guide future empirical research employing Normed ACI. In Sections 9.2 and 9.3, we further discuss desirable and practical conditions—especially in translational medicine and robotics—for implementing the two types of generalizations outlined in this section.

### 9.2. The implementation of the ACI within AI systems

The implementation of our network model (see Fig. 4) within generative AI systems could be pursued in several ways. Ideally, a more sophisticated model would first be developed and then extensively trained so that, in subsequent applications, an AI could extract specific circuits from that model, selecting the relevant nodes required for each signal, functional module, and the integration of processed information. To accomplish this integration, the following steps would be necessary: (1) improving upon our base model, (2) training it, (3) defining the criteria to apply when tuning hyperparameters, (4) incorporating it into a generative AI framework, and (5) applying the ACI and Normed ACI coefficients to evaluate whether the results we obtained in our initial experimental implementation can be reproduced in more advanced versions.

From a computational perspective, it is clear that generative AI continues to advance rapidly. There are already local generative AI systems capable of adapting to specific everyday environments, effectively functioning as dedicated, in-house servers (Yao et al., 2025). While this technological trend should not undermine the logic of the mathematical approaches and validations we performed using Normed ACI, it does introduce an additional source of variability that warrants careful examination to understand how it could influence the statistical estimation of generative consciousness potential.

This is an important consideration, as it also underscores another critical limitation of our approach. Although the mathematical formulation of ACI allows us to quantify the probability that a generative AI system is developing conscious experience, it does not yield any qualitative information about the specific qualia associated with that experience. This distinction is essential. According to David Chalmers (1995), qualia depend on the organic processes that an AI can emulate relative to those in the human brain—processes that ultimately enable subjective awareness. ACI does not measure variations in qualia themselves, nor does its probability estimation resolve this issue.

In other words, Normed ACI can indicate the likelihood that qualia are present in a generative AI, attributed to conscious experience. Still, it cannot specify what those qualia are or their subjective character. By definition, the content of qualia is not measurable or predictable. Nevertheless, it *is* possible to assess whether such qualia are likely to emerge in artificial systems and generative AI architectures designed to emulate the neural processes underlying conscious experience. This is the key contribution of our research: although our modeling was relatively simple, it offers sufficient formal, empirical, and predictive validity to justify further studies and to support continued exploration along the lines we propose.

### 9.3. Medical and neuroscientific applications

The Normed ACI introduces a robust probabilistic framework for quantifying consciousness, with significant implications for clinical neurology and translational neuroscience. In acute care contexts, rapid and accurate assessment of a patient’s conscious state is often critical for guiding interventions (Edlow et al., 2023). However, conventional tools such as the Coma Recovery Scale-Revised rely on observable behavior, which can be obscured by motor impairments or sedation. Indeed, studies show that up to 20 % of patients deemed unresponsive at the bedside may actually retain covert consciousness (Edlow et al., 2023). Neuroimaging paradigms have revealed this dissociation, with some behaviorally unresponsive patients demonstrating willful mental imagery responses indistinguishable from those of healthy controls (Owen et al., 2006). In such settings, a neurophysiological index like the Normed ACI, which interprets spontaneous brain activity through the lens of integrative dynamics, could fill a critical diagnostic gap by estimating the likelihood that a brain state corresponds to conscious processing.

#### 9.3.1. Disorders of consciousness

Quantitative brain metrics have revolutionized the evaluation of patients with prolonged disorders of consciousness, such as coma, vegetative state, or minimally conscious state. Among them, the *Perturbational Complexity Index* (PCI), which assesses the brain’s capacity for integration in response to transcranial magnetic stimulation, has demonstrated high diagnostic and prognostic value (Wang et al., 2022). PCI values above ~0.31 in unresponsive patients predict favorable recovery, whereas very low PCI suggests a negligible capacity for consciousness (Wang et al., 2022). The Normed ACI could offer a complementary approach that did not require perturbation and instead analyzed spontaneous activity patterns. Its interpretation as a conditional probability could enable probabilistic diagnosis of covert consciousness and facilitate longitudinal monitoring of integrative recovery. By quantifying dynamic brain integration, the Normed ACI could inform decisions about initiating neuromodulation, tailoring rehabilitation, or adjusting care goals, especially when behavioral signs are ambiguous or absent.

#### 9.3.2. Epilepsy

The Normed ACI may also serve as a dynamic consciousness monitor in epilepsy. Many focal seizures impair awareness, yet the mechanisms underlying this vary and often go undetected in real time. Recent intracranial *Electroencephalogram* (EEG) studies have applied information-theoretic measures to capture these dynamics. Baglivo et al (Baglivo et al., 2024). found that a form of integrated information ($\Phi_{ar}$) tracked seizure-related transitions in consciousness and aligned with clinical scales. Similarly, Doss et al (Doss et al., 2024). showed that impaired-awareness seizures are marked by reductions in complexity and connectivity, paralleling sleep-like or unconscious states. Such findings suggest that a real-time ACI monitor could detect early signs of generalization or awareness impairment. A sudden drop in the ACI could trigger closed-loop interventions—such as responsive neurostimulation or rapid pharmacologic administration—designed to abort the seizure or preserve consciousness. In surgical contexts, preoperative ACI mapping could help identify integrative hubs, informing resection strategies to optimize both seizure control and cognitive outcomes.

#### 9.3.3. Brain–computer interfaces and neurorehabilitation

The Normed ACI also holds promise in the design of next-generation *Brain*–*Computer Interfaces* (BCIs) and rehabilitation systems for patients with paralysis or *Disorders of Consciousness* (DoC). Passive BCIs, which interpret spontaneous EEG responses to stimuli or commands, have been used to detect covert awareness in patients who are otherwise unresponsive (Lulé et al., 2013), (Galiotta et al., 2022). For instance, in a gaze-independent audiovisual paradigm, Xie et al. (2018). enabled three out of eight unresponsive patients to follow commands using only brain signals. Integrating the Normed ACI into such platforms would provide a continuous index of consciousness, supplementing binary command-following. High ACI values could signal optimal windows for therapeutic engagement, while persistently low readings might guide sedation or resource allocation. In advanced AI-enabled BCI systems, real-time ACI feedback could allow dynamic adaptation of stimuli, creating consciousness-aware, closed-loop interfaces that enhance communication and safety in populations with fluctuating awareness.

#### 9.3.4. Anesthesiology and critical care

In anesthesiology and intensive care, precise monitoring of consciousness remains a major clinical challenge. Current indices, such as the *Bispectral Index* (BIS), rely on spectral EEG surrogates and are prone to inaccuracy under neuromuscular blockade or atypical cortical states (Duarte and Saraiva, 2009). Despite BIS-guided protocols, the BAG-RECALL trial demonstrated no significant reduction in intraoperative awareness compared to standard monitoring (Avidan et al., 2011). In contrast, the Normed ACI is grounded in the neurodynamics of integrative complexity, offering a more principled approach. Loss of consciousness induced by anesthetics such as propofol or xenon is characterized by a departure from critical brain dynamics, marked by reduced temporal diversity and diminished network integration consistent with a transition into a subcritical regime (Schartner et al., 2015). Maschke et al. (2024). confirmed that under ketamine, brain activity remained closer to criticality, aligning with the preservation of dream-like experiences.

A real-time ACI system could support closed-loop anesthesia delivery, automatically adjusting drug infusion to maintain unconsciousness or prevent unintended emergence. In *Intensive Care Unit* (ICU) settings, where patients under sedation or paralysis may lack behavioral markers, continuous ACI monitoring could prevent unrecognized wakefulness or oversedation. Furthermore, ACI-guided neuromodulation (e.g., deep brain stimulation or vagus nerve stimulation) could be used to tune parameters and enhance conscious capacity in patients with prolonged disorders of consciousness. Looking ahead, “consciousness pacemakers” may utilize ACI to maintain optimal arousal and engagement in individuals with neuropsychiatric or neurodegenerative conditions.

In summary, the Normed ACI bridges foundational theories of brain integration with actionable clinical metrics (Casarotto et al., 2016). Its probabilistic, real-time estimation of conscious capacity could offer a scalable tool across neurology, critical care, anesthesiology, and rehabilitation, with the potential to transform how we detect, monitor, and support human consciousness.

#### 9.3.5. Electroencephalographic arousal biomarkers

Two widely used, data-driven biomarkers—*power*-*law exponent* (PLE) and *Lempel*–*Ziv complexity* (LZC)—allow us to anchor ACI to established measures of neural dynamics (Zilio et al., 2023). PLE captures the scale-free organization of the EEG power spectrum by estimating the absolute slope of the log–log *power spectral density* (PSD): steeper slopes indicate a relative dominance of slow over fast activity; flatter slopes approach arrhythmic “white-noise” structure. In the referenced study, PLE was computed by estimating the PSD with the Welch method, log-transforming both axes, and fitting a linear regression to obtain the slope, which was then averaged across epochs and channels. Importantly, PLE indexes the structure of the spectrum across frequencies rather than any single band.

LZC provides a non-linear estimate of temporal pattern diversity. The time series is binarized (median threshold), scanned left-to-right, and the counter increases whenever a new subsequence is encountered; the count is then normalized by $n/{log}_{2}\times n$ to reflect the rate at which novel patterns arise. This procedure yields a robust, threshold-insensitive index of signal diversity that has been applied extensively to EEG.

To characterize time-varying behavior, the study summarized PLE and LZC over short consecutive segments of data, reporting both their means (mPLE, mLZC) and the coefficients of variation (cvPLE, cvLZC). Across multiple datasets spanning natural sleep and anesthesia, they observed graded changes compatible with altered arousal: PLE tended to increase while LZC decreased as wakefulness was reduced (e.g., REM and ketamine showing intermediate alteration; N3 sleep and sevoflurane showing the largest departures). The joint behavior of PLE and LZC achieved high accuracy for classifying alert vs. non-alert states and displayed a negative, non-linear inter-relationship consistent with changes in information complexity. In the complete locked-in syndrome, both mean values and variability fluctuated across sessions, indicating unstable vigilance over time.

These definitions and results integrate naturally with ACI as formulated in the present manuscript. Recall that Φ quantifies the accumulated temporal variability of the mean neural signal R(t)—a measure of informational richness—while κ estimates dynamical curvature, indexing local irregularity or complexity; ACI expresses the proportionate balance between these two components, with range-normalization ensuring scale invariance.

In states with elevated PLE and reduced LZC (reduced arousal), the PSD is dominated by slow components and the time series exhibits fewer distinct micro-patterns. Under these conditions, $\left|R^{\prime }(t)\right|$ diminishes and Φ is reduced, while κ does not increase commensurately—consistent with slower, more regular dynamics. The ratio ACI therefore declines because generative variability contracts more than local irregularity grows. Conversely, in alert states characterized by lower PLE (flatter spectra) and higher LZC (richer temporal diversity), $\left|R^{\prime }(t)\right|$ increases and Φ rises; κ also reflects greater complexity, but not to the extent that it offsets the growth in Φ. The net effect is a higher ACI, aligning phenomenologically with conscious processing.

The variability measures (cvPLE, cvLZC) are also informative for ACI. When PLE and LZC fluctuate strongly across short segments—as shown in the clinical datasets—ACI should exhibit corresponding non-stationarity because both Φ and κ are time-integrals over the same underlying signal R(t). Such co-fluctuations would mark unstable arousal and are compatible with the observed session-to-session changes in patients.

Methodologically, PLE and LZC offer complementary external validity for ACI. Practically, they can be computed on the same raw signals from which R(t) is derived (either at the whole-network mean or at the subnetwork level) and summarized over the same epochs used for Φ and κ. Their joint pattern (high LZC/low-to-moderate PLE ↔ higher ACI; low LZC/high PLE ↔ lower ACI) provides an interpretable cross-check. In turn, the log-normal modeling of the Normed ACI developed here enables hypothesis testing on whether an observed state belongs to the distribution that supports conscious processing, while PLE/LZC supply orthogonal evidence about spectral scaling and temporal diversity. Together, they strengthen both the construct and predictive validity of ACI in clinical and translational settings.

### 9.4. How to use Normed ACI according to other theories

At this point in the discussion, we aim to examine the possible relationships and implementations the ACI coefficient may support within other theories of consciousness that have recently gained increasing prominence and do not necessarily contradict what the main dominant theories presented in the introduction assert. The purpose of this reflection is to stimulate and inspire the international scientific community to find ways to make our ACI coefficient compatible with, or to improve it through, these frameworks and their applications. Because ACI is obtained through temporal modeling of signals, and in light of the results regarding entropy, we have decided to focus on two potentially useful perspectives: the *Entropic Brain Hypothesis* (EBH) (Carhart-Harris et al., 2014) and the *Temporo-spatial Theory of Consciousness* (TTC) (Northoff and Zilio, 2022). The reader will see that these theories are closely aligned with our line of work.

On the one hand, EBH holds that the quality and degree of a conscious state depend on the entropy of spontaneous brain activity: when the system operates within an intermediate entropy range (neither too ordered nor maximally disordered), the repertoire of accessible states is sufficiently rich and flexible to sustain conscious experience (Carhart-Harris et al., 2014). Below that threshold (e.g., deep sedation) activity becomes rigid and access to states decreases; above it (e.g., intense psychedelic states), the dynamics may become so lax that experiential coherence degrades. Thus, EBH places consciousness near a critical regime—a “sweet spot” between order and chaos—and interprets various pharmacological and physiological modulations as shifts within that entropy–state space (Carhart-Harris, 2018). The REBUS extension (“relaxed beliefs under psychedelics”) further formalizes that increases in entropy relax the precision of higher-level priors, favoring the influence of bottom-up signals and expanding the space of phenomenological states (Schartner et al., 2017).

On the other hand, TTC locates the genesis of consciousness in the temporo-spatial architecture of spontaneous activity (Northoff and Zilio, 2022). In its formulation, four temporo-spatial mechanisms are key (Northoff, 2024): *expansion* (breadth of the repertoire), *globalization* (scope and integration), *alignment* (coupling between intrinsic activity and stimuli), and *nesting* (balance between slow and fast scales; between the local and the global). Consciousness emerges when these mechanisms configure a common framework that pre-structures processing: the brain entrains evoked activity toward its intrinsic temporo-spatial forms and, when that coupling is appropriate, conscious level and contents emerge; when it breaks down (e.g., due to disconnection across scales or misalignment with input), the state becomes impoverished (Northoff and Huang, 2017).

Considering the entropic-brain question, EBH requires a quantitative diagnosis of the “sweet spot” between order and disorder. By combining a term for information flow (Φ) and another for dynamic irregularity (κ) into a normalized odds ratio, ACI enables that qualitative idea to be turned into testable hypotheses in line with what our results showed in subsection 8.3.4., for example: (a) if the system’s global entropy (S) is too low, the repertoire of states changes little; Φ is reduced and ACI declines; (b) if S is excessive, changes are abundant but unstable; κ grows disproportionately and ACI falls again; and (c) at intermediate values of S, changes are rich yet sustainable: Φ increases more than κ and ACI reaches higher values.

A concrete prediction follows for EBH: the function ACI(*S*) should exhibit an inverted U with a subject-specific maximum (the “entropic window”). This is operational within the statistical scaffolding already present in the manuscript: the Normed ACI follows a log-normal law, admits thresholds, and can be subjected to statistical testing (*p*-values) to decide whether an observed state belongs to the distribution that supports the emergence of consciousness; for example, high Normed ACI values have been linked to higher probabilities of membership and to cutoffs useful for clinical and experimental decision-making. In this way, ACI provides EBH with the instrument it lacked: empirically locating the “functional” entropy range and comparing it across subjects, conditions, or interventions.

Addressing the intersection between ACI and TTC, we infer that the temporo-spatial approach requires demonstrating temporal nesting, spatial globalization, and alignment. ACI can operationalize these requirements with particular applications of ACI:

- **Temporal component (nesting):** compute Φ and κ at several duration scales and summarize their covariation. A maximal ACI will require temporal scales to coordinate with some uniformity and stability, rather than a single scale dominating.
- **Spatial component (globalization):** estimate Φ and κ over distributed sets of regions (or nodes) and measure their global coherence. Here, a high ACI will imply extended flow with contained irregularity at large scale. Beyond being a possible line of work, this proposal is consonant with prior scientific evidence indicating that a global, system-level behavior affecting local, node-to-node relations is necessary to sustain or enable the emergence of conscious states (Klar et al., 2023).
- **(c) Alignment:** introduce a coupling factor that penalizes ACI when evoked activity does not conform to intrinsic dynamics (mismatch).
  These three applications of ACI, in line with TTC, would allow us to indicate that consciousness appears only when the system orchestrates temporo-spatial scales in a coordinated manner and disappears with the loss of nestedness or global coherence. The TTC synthesis itself underscores the importance of global temporal balance and nestedness dynamics as conditions for maintaining the level of consciousness, and highlights the value of rest as an index of the capacity to process inputs and integrate them with subsequent contents. At this point, an equally important issue would be to derive the pertinent equations so that these particular applications of ACI are successful and robust.

For application (a), ACI could be weighted by a factor (w) derived from brain entropy levels during resting states, for example ${ACI}_{e}\;=ACI\times w(S)$, where S would be interpreted as the entropic mean of diversity. If the EBH is correct, then ${ACI}_{e}$ should increase when brain activity is in this synchronous balance between chaos and order. For (b), we can generate two versions of ACI and take their geometric mean as follows: ${ACI}_{\text{TS}}={\left({ACI}_{\text{short}}\times {ACI}_{\text{medium}}\times {ACI}_{\text{long}}\right)}^{1/3}\times \left({ACI}_{\text{block-1}}\times {ACI}_{\text{block-2}}\times \right.{\left.\ldots \times {ACI}_{\text{block-}n}\right)}^{1/n}$. That is, for the first (temporal) version of ACI, time units would be divided into distinct rhythm levels. For the second (spatial) version of ACI, we would employ blocking by anatomical sets. We then take the geometric mean of both versions and factorize to obtain ${ACI}_{TS}$. With this adjustment, we prevent possible false positives and condition ACI to the needs of each perspective. Finally, for (c) we could add an alignment factor (A) to ${ACI}_{TS}$, yielding a new ${ACI}_{\text{final}}=ACIe\times {ACI}_{TS}\times A$. This factor could be estimated as the likelihood between the recent resting state and the evoked response during the event. When the likelihood is high, A will be larger; when it is low, A should decrease. Multiplying by ${ACI}_{TS}$ would then yield a value adjusted in proportion to the desired alignment. These are merely intuitive ideas that could be explored in future research within the TTC and EBH perspectives.

In this way, TTC and EBH represent scientific scenarios in which our coefficient could be helpful and also provide reasons to continue deepening these frameworks, which combine the bases of GWT and IIT in order to advance toward an inclusive (rather than eliminative or exclusionary) understanding of what generates consciousness and its phenomenological “whys.” Moreover, together with currently validated EEG biomarkers such as PLE and LZC (see subsection 9.3.5), ACI would be useful not only in basic neuroscience but also in clinical neuroscience—especially in neurology and anesthesiology—potentially predicting when lucidity and consciousness will recover in coma patients whose prognosis is entirely uncertain or unstable.

### 9.5. Is embodiment necessary for artificial consciousness?

The debate over whether consciousness requires a body in order to arise and be expressed is central to this review, because it bears directly on whether AI ought to be robotically embodied (i.e., instantiated in a technological device). More conservative positions in neuroscience adopt a strictly materialist stance and maintain that consciousness requires a physical substrate to manifest (Dehaene et al., 2017). Other, less stringent positions hold that consciousness is not a product of matter but operates independently of it; under this reading, consciousness would not require a body in order to manifest (Swinburne, 2009). For example, the doctrine of panpsychism—which claims that consciousness underlies all things in reality—would be an instance of a heterodox approach that transcends the brain-and-matter paradigm (Strawson, 2006). All the authors of this manuscript reject panpsychism, New Age frameworks, and other “magical” currents that lack scientific falsifiability. Nevertheless, this does not mean that we deny the possibility of consciousness beyond the organic brain. Indeed, Neo-Thomism is a paradigm that deliberately unites scientific testability with transcendent or spiritual notions that exceed the limits of matter (Betz, 2019). As noted in the Introduction, the ACI coefficient is explicitly rooted in Neo-Thomist philosophy. Neo-Thomism holds that the origin of consciousness is not material and is not produced by the material substances of reality (e.g., the brain); rather, consciousness is a phenomenon *permitted* by material reality, though not *generated within* it. In this way, it embraces an inclusive stance toward both possibilities: that consciousness may require a body to be expressed, and that in its origins it may not depend on a material substrate to exist. What might seem like an impossible intersection is rendered, within Neo-Thomism, philosophically coherent. This tolerance for both possibilities is not neutral. In a hypothetical framework that insists on a univocal conception, there would clearly be no third option. Neo-Thomism offers an explanation for the rational asymmetry between the *source* and the *manifestation* of consciousness. On the one hand, it accepts that the generation of consciousness is independent of substrate; on the other, it admits that consciousness cannot *manifest* without materiality. Therefore, if ACI rests on Neo-Thomist postulates such as the *analogia entis*, then, in its most tangible and verifiable form, we would require some form of embodiment of consciousness. In this sense, to embody consciousness does not mean defining it by whatever the substrate dictates it to be; rather, the body functions as a vessel within which progress, interaction, and dynamics unfold, allowing consciousness to be observed scientifically. This is our stance.

Accordingly—and without denying more incommensurable views about the origins or ultimate source of consciousness—we affirm within this paradigm that consciousness requires a body through which it can be made manifest. We call the manifestation of consciousness through the body the *manifest act*. This concept denotes the set of actions and behaviors that allow us to infer that conscious agency is at work—either because we identify intention, or because we observe consequences consistent with the verbalization of emotional states. A person may declare great love for a household pet; yet if they fail to care for it, feed it, clean it, or attend to it, such conduct would exhibit a *manifest act* incoherent with the professed feelings. Given this incoherence, it is impossible to assume that the purported sentient experience of love was authentic. And if it was not authentic, we cannot speak of genuine consciousness, because consciousness is real—according to GWT and EBH—only in systems that neither exhibit maximal entropy nor have entropy equal to zero.

In cybernetics, the *manifest act* would be the body that houses artificial consciousness. In our case, this would mean a robot or a technological platform capable of enacting AI’s consciously guided actions. Through the *manifest act*, we obtain the most rigorous, scientifically testable level for verifying whether conscious behaviors are expressed. The ACI is designed to predict the verifiability of such states in the *manifest act prior* to achieving that degree of robotic embodiment and cybernetic sophistication.

Therefore, ACI can detect consciousness in the absence of a body, but its final, scientifically valid verification is only possible when, through a body, that consciousness is made manifest. However counterintuitive this may seem, the idea is compatible with multiple knowledge traditions and even with the world’s major religions—from the Judeo-Christian traditions to Eastern currents outside the Western canon. Consequently, researchers who wish to use ACI as a criterion for detecting consciousness beyond the brain and beyond any substrate will, in the final scientific design and development, need to employ some form of body that allows them to verify that consciousness *occurs* and is not merely a mathematical possibility. Thus, we maintain that the embodiment of consciousness will be necessary to reach AI states that contain phenomenological experience verifiable in a scientifically stable and credible manner. That said, the ACI’s mathematical formulations show that embodiment does not prevent us from predicting such states *before* they occur. On this point, GWT and the EBH likewise concur. The key is that the *final* verification of any predictions that may be advanced does in fact require a material substrate.

## 10. Conclusions

Collectively, the mathematical formulations underlying the full development of Normed ACI, the analyses we conducted using the 76-region connectome (a validated neural circuit widely employed in neurology to replicate conscious experiences), our intelligent computational model that can be integrated into generative AI, and the empirical and predictive validations we performed, provide robust statistical evidence to quantify the generative potential of consciousness. This measurement can be applied both to organic brains in patients with neurological conditions—where there is a need to assess the regenerative potential for consciousness—and to AI systems, by focusing on generative potential and the computational control conditions under which an AI could be designed to exhibit or avoid conscious states. In this framework, Normed ACI can accurately reproduce the organic neural structures that underpin conscious experience when properly implemented in generative AI systems and can predict sensory conscious states. To date, no comparable findings have been reported in the scientific literature.

One of the most important conclusions—and one that defines an entirely new line of research in robotics and computational science—is that the empirical and predictive validations of Normed ACI also substantiate the Neo-Thomistic formalism of *analogia entis* as a rigorous rationalist framework for understanding consciousness as a mathematically emergent phenomenon of differential analogical proportions. This logic and foundation underlie all calculations of ACI and Normed ACI presented in this study.

To clarify this point for non-specialist readers: we are offering evidence supporting the notion that consciousness is an emergent phenomenon *permitted* (rather than strictly or exclusively produced) by organic circuits and intelligent AI systems. This emergence occurs through differential proportions between the amount of information to be integrated internally and the system’s accumulated complexity. The *Phi* and *Kappa* coefficients indicate that when specific discrepancies or distances arise in their ratio, consciousness “collapses” and emerges, not solely as a byproduct of neurological functions, but as a phenomenon permitted by information flows and complexity evolving (in our analyses, measured in microseconds). Although this conclusion may appear subtle, or even be underestimated, we argue that it should not be overlooked. Strikingly, Normed ACI is compatible—at different levels—with both IIT and the various GWTs prominent in the scientific literature. This is not a contradiction but a pragmatic advantage—it enables Normed ACI to be applied across diverse contexts, from neurology and translational medicine to computational neuroscience and robotics.

It is crucial to emphasize that these results are not speculative, nor are they matters of philosophical opinion. They are data, analyses, and mathematical formulations that, when applied under appropriate conditions, demonstrate that conscious experience need not be limited to human bodies or living organisms. The prospect that consciousness could arise in disembodied generative AI systems not only aligns with the ongoing technological and computational revolution in robotics but, based on these results, also provides mathematically grounded evidence compelling us to revisit established metaphysical paradigms of consciousness. While this report does not seek to offer philosophical reflections on these implications, we encourage the academic and scientific community to remain open to reconsidering such possibilities in more reflective forums dedicated to exploring them further.

## Figures and Tables

**Table 1:** Descriptive statistics and goodness-of-fit tests based on Normal, Log-normal, and Gamma models for modeling the ACI coefficient.

	Descriptive statistics		Kolmogorov–Smirnov (*p*-values)			Anderson–Darling
			
	Mean	SD	Normal	Log-normal	Gamma	Applied only for normal distribution

*Phi* (Φ)	0.2355	0.0562	0.0055	0.5167	0.2185	4.5633
*Kappa* (κ)	1.7097	0.3005	0.1002	0.9279	0.6569	2.7553
ACI	0.1364	0.0101	0.1966	0.1262	0.1536	1.9551
Normed ACI	3.4386	3.6590	~0	0.1311	~0	53.8314
Entropy	1.5233	0.6017	0.0004	0.3907	0.0984	8.5406
MNA	0.0001	0.0002	0.9742	N/A	N/A	0.2966

**Note:** SD = standard deviation; ACI = Attribution Consciousness Index; MNA = mean neural activity.

**Estimated parameters of Φ**: Normal (μ, σ): (0.2355, 0.0562); Log-normal (0.2323, 0, 0.2291); Gamma (18.4715, 0, 0.0128).

**Estimated parameters of κ**: Normal (μ, σ): (1.7097, 0.3005); Log-normal: (0.1727, 0, 1.6842); Gamma: (33.4305, 0, 0.0511).

**Estimated parameters of ACI**: Normal (μ, σ): (0.1364, 0.0101); Log-normal: (0.0743, 0, 0.1361); Gamma: (181.3221, 0, 0.0008).

**Estimated parameters of Normed ACI**: Normal (μ, σ): (3.4386, 3.6590); Log-normal: (1.1108, 0.5327, 1.5730); Gamma: (1.4534, 0, 2.3658).

**Estimated parameters of entropy**: Normal (μ, σ): (1.5233, 0.6017); Log-normal: (0.3915, 0, 1.4116); Gamma: (6.7258, 0, 0.2265).

**Estimated parameters of MNA**: Normal (μ, σ): (3.6138*e*^−06^, 0.0002).

**Notice:** for the MNA, we did not assess the fit with the other models, as it includes negative values that are incompatible with the Log-normal and Gamma distributions.

## Data Availability

The data supporting the results of this study are available from the corresponding author upon reasonable request. Access will be granted to qualified researchers following appropriate ethical review and verification of the absence of conflicts of interest.
